# Netrin 1 directs vascular patterning and maturity in the developing kidney

**DOI:** 10.1242/dev.201886

**Published:** 2023-11-24

**Authors:** Samuel E. Honeycutt, Pierre-Emmanuel Y. N'Guetta, Deanna M. Hardesty, Yubin Xiong, Shamus L. Cooper, Matthew J. Stevenson, Lori L. O'Brien

**Affiliations:** Department of Cell Biology and Physiology, University of North Carolina at Chapel Hill, Chapel Hill, NC 27599, USA

**Keywords:** Netrin 1, Vasculature, Kidney development, Stroma, Signaling, Patterning, Mouse

## Abstract

The intricate vascular system of the kidneys supports body fluid and organ homeostasis. However, little is known about how vascular architecture is established during kidney development. More specifically, how signals from the kidney influence vessel maturity and patterning remains poorly understood. Netrin 1 (Ntn1) is a secreted ligand that is crucial for vessel and neuronal guidance. Here, we demonstrate that *Ntn1* is expressed by Foxd1^+^ stromal progenitors in the developing mouse kidney and conditional deletion (*Foxd1^GC/+^;Ntn1^fl/fl^*) results in hypoplastic kidneys with extended nephrogenesis. Wholemount 3D analyses additionally revealed the loss of a predictable vascular pattern in *Foxd1^GC/+^;Ntn1^fl/fl^* kidneys. As vascular patterning has been linked to vessel maturity, we investigated arterialization. Quantification of the CD31^+^ endothelium at E15.5 revealed no differences in metrics such as the number of branches or branch points, whereas the arterial vascular smooth muscle metrics were significantly reduced at both E15.5 and P0. In support of our observed phenotypes, whole kidney RNA-seq revealed disruptions to genes and programs associated with stromal cells, vasculature and differentiating nephrons. Together, our findings highlight the significance of Ntn1 to proper vascularization and kidney development.

## Teaser

**Summary:** Through genetic deletion, we disrupt netrin 1 signaling in the developing kidney and uncover previously unreported roles in mediating vascular patterning and maturity and proper kidney development.

## INTRODUCTION

The organization of blood vessels into a network capable of maintaining organ and body fluid homeostasis requires both proper development of the vasculature and specialization unique to the tissue. Inappropriate vascular formation and patterning can result in pathologies in many organs (Blei and Bittman, 2016; Heimann and Damato, 2010; Queisser et al., 2021; Wälchli et al., 2023). Vascular endothelial cells organize into branched, hierarchical networks that form a continuous system for blood flow. Larger arteries branch into smaller arterioles, which further divide into capillaries. Venules then return the blood to larger veins and continue its circulation (Potente and Mäkinen, 2017). The vessels within these networks are distinct in their morphology and function, which is often tailored to the precise needs of the organ. For example, the endothelium of the kidney glomerular capillaries and ascending vasa recta are fenestrated to enable their unique functions in filtration and diffusion, respectively (Molema and Aird, 2012; Pallone et al., 2003). In association with their functions, the endothelium of these different vascular beds also has distinct molecular signatures (Barry et al., 2019; Daniel et al., 2018). In addition, endothelial association with mural cells delineates the various types of vessels within an organ. Larger vessels such as arteries are coated by vascular smooth muscle cells (vSMCs) whereas smaller capillaries are covered by pericytes, with each type of mural cell supporting distinct vascular functions (Potente and Mäkinen, 2017). Altogether, these vascular properties highlight the need for precise developmental programs that support the proper formation of these complex networks.

Recent studies have begun to shed light on the progressive formation of kidney vascular networks during development. The temporal progression and predictable rudimentary patterning of the major renal arteries through mid-gestation were elucidated through comprehensive imaging studies (Daniel et al., 2018; Munro et al., 2017). Arteries which connect to the aorta undergo angiogenic growth towards the developing kidney, forming a vascular ring around the base of the ureteric bud by embryonic day (E) 11.25 (Munro et al., 2017). Continued growth, remodeling and maturation of the endothelial network leads to the establishment of an arterial tree by E13.5 that forms a predictable pattern (Daniel et al., 2018). Renin-expressing cells are localized along the developing arterial vasculature and mark points of branching, and renin itself is important for proper vascular development (Gomez et al., 1989; Reddi et al., 1998; Sequeira-Lopez et al., 2015b; Takahashi et al., 2005). At the molecular level, vascular patterning was shown to be regulated by the association of mural cells with the nascent endothelium and modulated in part by Pbx1 (Hurtado et al., 2015). Deletion of the transcription factor *Pbx1* from the Foxd1^+^ stromal progenitors induces precocious differentiation and premature association of vascular mural cells with the endothelium, leading to stochastic arterial patterning and impaired renal function (Hurtado et al., 2015). A separate report showed that Dicer1 activity in the kidney stroma is important for patterning smaller vessels, such as the peritubular and glomerular capillaries (Nakagawa et al., 2015). Although these studies have garnered some of the first insights into how the kidney vascular network is established and organized, we still lack a clear understanding of how this process is modulated at the molecular level.

Signaling molecules play important roles during development, often providing instructional cues which help pattern the developing tissues. In respect to vasculature, a number of signaling pathways, which also regulate axon guidance, provide angiogenic guidance. This includes Slit/Robo, Eph/Ephrin, Semaphorins/Plexin-Neuropilins, and Netrins/UNC5 (Adams and Eichmann, 2010). These guidance cues direct vascular development during embryogenesis and, unsurprisingly, the removal of many of these cues or their receptors results in vascular defects and mortality in the forming embryo (Bin et al., 2015; Gerety et al., 1999; Gitler et al., 2004; |Gu et al., 2005; 2003; Jones et al., 2008; Lu et al., 2004). Netrin 1 (Ntn1) is a classical neuronal guidance cue well-known for its role in commissural axon guidance (Kennedy et al., 1994; Serafini et al., 1994). However, roles for Ntn1 have expanded to include mediating pancreas development, inner ear morphogenesis, mammary gland development, lung branching and angiogenesis (Larrivée et al., 2007; Liu et al., 2004; Lu et al., 2004; Salminen et al., 2000; Srinivasan et al., 2003; Sun et al., 2011; Wilson et al., 2006; Yebra et al., 2003). Endothelial guidance by Ntn1 is accomplished through chemotactic interactions with its cognate receptors in the vasculature, Unc5b and CD146 (Mcam), which regulate blood vessel growth, branching and patterning by promoting either a repulsive or attractive response, respectively (Larrivée et al., 2007; Lu et al., 2004; Tu et al., 2015). Whether Ntn1 plays a similar role in guiding vascular development and patterning in the kidney remains unexplored.

Here, we report that *Ntn1* is expressed and secreted by stromal progenitors in the developing kidney. Deletion of *Ntn1* from this population results in hypoplastic kidneys and delayed cessation of nephrogenesis. In correlation with its known role in vascular development, we found that Ntn1 supports proper patterning and maturation of the vasculature during kidney organogenesis. Our findings correlate with those in the accompanying article by Luo et al. (2023) and together we establish a crucial role for Ntn1 in mediating proper kidney development through regulation of vascular maturity.

## RESULTS

### *Ntn1* is expressed and secreted by Foxd1^+^ stromal progenitors in the developing kidney

**Fig. 1.**
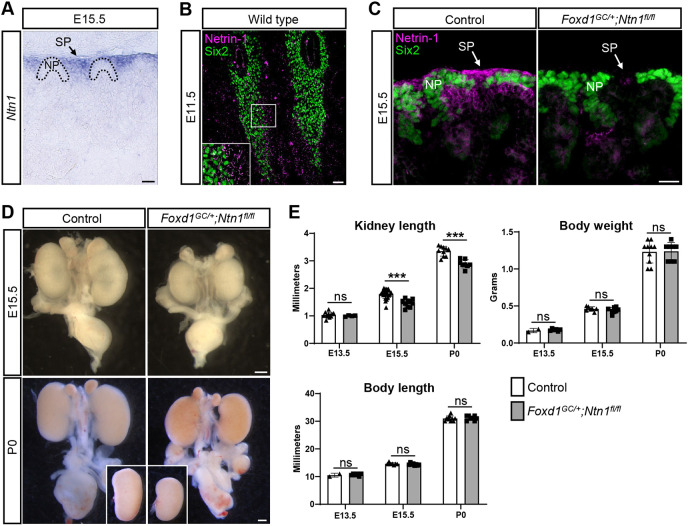
***Ntn1* is expressed and secreted by stromal progenitors in the kidney and *Ntn1* deletion results in kidney hypoplasia.** (A) E15.5 *in situ* hybridization section showing *Ntn1* expression in stromal progenitors (SP) surrounding nephron progenitors (NP). Dotted lines demarcate NP niches. (B) E11.5 kidney section immunostaining showing Ntn1 (magenta) secretion by stromal progenitors surrounding Six2^+^ (green) nephron progenitors. Inset shows higher magnification of Ntn1 signal adjacent to nephron progenitors. (C) Immunofluorescence images showing Ntn1 (magenta) signal in stromal progenitors adjacent to Six2^+^ nephron progenitors (green), as well as the ureteric tip region. Genetic deletion of *Ntn1* in the *Foxd1*^+^ stromal population (*Foxd1^GC/+^;Ntn1^fl/fl^*) results in a significant reduction in Ntn1 signal. (D) Kidney hypoplasia is observed in *Foxd1^GC/+^;Ntn1^fl/fl^* animals at E15.5 and P0. Whole urogenital systems are pictured. Insets for P0 show the same kidneys micro-dissected and side-by-side. (E) Kidney length, body length and body weight were measured at E13.5, E15.5 and P0 with the kidney size reduction in *Foxd1^GC/+^;Ntn1^fl/fl^* animals becoming statistically significant by E15.5. Graphs show mean±s.d. with points representing biological replicates. ***P≤0.001 (unpaired, two-tailed Student's *t*-test). ns, not significant. Scale bars: 50 µm (A,B); 25 µm (C); 500 µm (D).

Although netrin family members including Ntn1 play important roles in other branching organs during development, such as the lung, pancreas and mammary gland, whether they have a role in kidney development has not been investigated to date (Liu et al., 2004; Srinivasan et al., 2003; Yebra et al., 2003). To begin, we examined the expression pattern of *Ntn1* mRNA in the developing kidney and found that *Ntn1* is expressed by stromal progenitors around the nephron progenitor caps at E15.5 and, to a lesser extent, in the proximal tubule which is more specifically detected at later stages of development, at postnatal day (P) 2 (Fig. 1A; Fig. S1A). This cellular expression pattern is confirmed by publicly available single cell (sc) RNA-seq data of the kidney at E18.5 (Combes et al., 2019; England et al., 2020). As early as E11.5, secreted Ntn1 protein was observed surrounding the Six2^+^ nephron progenitors where the stromal progenitors coalesce (Fig. 1B). At E15.5, Ntn1 was similarly secreted into the stromal progenitor niche surrounding the nephron progenitors (Fig. 1C). Some protein was diffused into the area below the nephron progenitors where ureteric tips lie, which may be relevant to signaling or serve as a ligand sink to prevent further diffusion (Fig. 1C). The stromal progenitors expressing *Ntn1* give rise to renal interstitium, mesangial cells and vascular mural cells, among other cell types, and function in advancing nephron progenitor differentiation (Hatini et al., 1996; Kobayashi et al., 2014; Sequeira-Lopez et al., 2015a), suggesting that Ntn1 signaling may support proper kidney development.

### Deletion of *Ntn1* from stromal progenitors disrupts normal kidney development

Germline deletion of *Ntn1* was previously shown to result in embryonic lethality by E14.5 (Bin et al., 2015). To circumvent this early lethality and address the role of Ntn1 in kidney development, we conditionally deleted *Ntn1* from the Foxd1^+^ stromal progenitors by using the *Foxd1^GC^* allele in combination with an *Ntn1* floxed allele (Bin et al., 2015; Humphreys et al., 2010; Kobayashi et al., 2014). The resulting *Foxd1^GC/+^;Ntn1^fl/fl^* mice have very little or no Ntn1 protein present in the interstitial regions surrounding the nephron progenitors, confirming efficient deletion (Fig. 1C). Upon analysis of overall gross morphology, we found that *Foxd1^GC/+^;Ntn1^fl/fl^* animals have hypoplastic kidneys at E15.5 and this phenotype persists postnatally (Fig. 1D). Immunostaining of the ureteric tree at E14.5 also revealed qualitative differences in kidney size (Fig. S1B). Measurements of kidney length showed these reductions became most significant after E13.5, whereas body weight and body length remained comparable, with no significant differences between controls and *Foxd1^GC/+^;Ntn1^fl/fl^* animals (Fig. 1E).

**Fig. 2.**
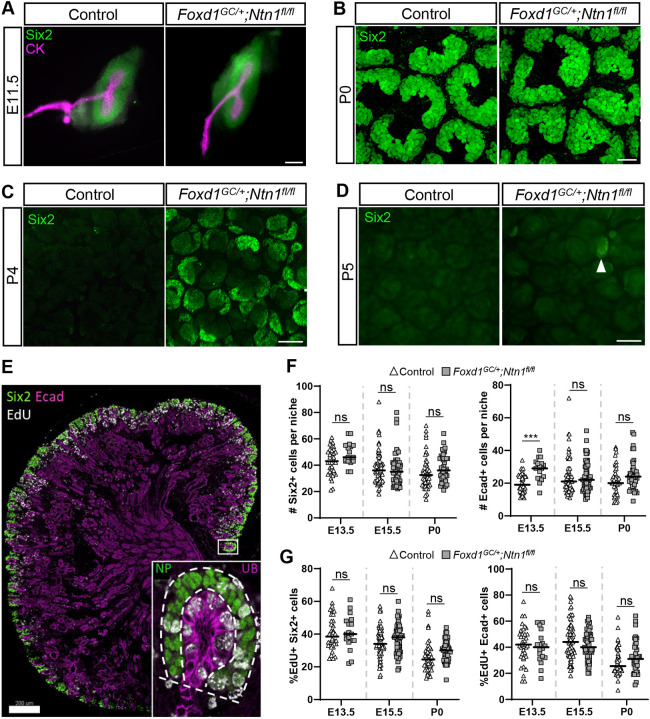
**Loss of stromal-derived Ntn1 delays the cessation of nephrogenesis without significant differences in progenitor parameters.** (A) Whole kidney immunostaining at E11.5 shows that kidney development is initiated normally in *Foxd1^GC/+^;Ntn1^fl/fl^* animals with the expected T-shaped ureteric bud (cytokeratin, CK, magenta) surrounded by nephron progenitors (Six2, green). (B) *Foxd1^GC/+^;Ntn1^fl/fl^* nephron progenitor niches (Six2, green) appear to be normally organized at P0 in surface views of wholemount immunostained kidneys. (C) Wholemount immunostains of P4 kidneys show the persistence of Six2^+^ nephron progenitors (green) on the surface of *Foxd1^GC/+^;Ntn1^fl/fl^* kidneys. (D) Wholemount immunostains at P5 show that nearly all Six2^+^ nephron progenitors (green) in *Foxd1^GC/+^;Ntn1^fl/fl^* kidneys have differentiated. White arrowhead highlights a small pool of remaining Six2^+^ cells. (E) Example of a sectioned kidney labeled with EdU (white) and immunostained with Six2 (green) and E-cadherin (Ecad, magenta) to label the nephron progenitors and collecting duct system, respectively. Inset shows high magnification image of a niche with dotted lines highlighting what was considered a nephron progenitor (NP) niche or ureteric bud (UB) tip niche for quantification purposes. (F) Quantification of Six2^+^ nephron progenitors or Ecad^+^ ureteric tip cells at E13.5, E15.5 and P0 shows no significant differences in *Foxd1^GC/+^;Ntn1^fl/fl^* total cell numbers except for Ecad^+^ cells at E13.5. (G) Quantification of the percentage of EdU^+^ Six2^+^ nephron progenitors or EdU^+^ Ecad^+^ ureteric tip cells reveals no significant difference at E13.5, E15.5 or P0 between *Foxd1^GC/+^;Ntn1^fl/fl^* kidneys or controls. Graphs show mean±s.d. for three biological replicates, with points representing niches. ***P≤0.001 (unpaired, two-tailed Student's *t*-test). ns, not significant. Scale bars: 100 µm (A); 30 µm (B-D); 200 µm (E).

As a means of assessing whether *Ntn1* deletion affects either the onset of kidney development or cessation of nephrogenesis, we examined the presence of Six2^+^ nephron progenitors at the initial branching event (E11.5) and after the cessation of nephrogenesis (P4), which normally occurs at ∼P2-P3 in the mouse (Hartman et al., 2007; Self et al., 2006). At E11.5, we observed no empirical differences in the condensation of Six2^+^ nephron progenitors around the ureteric buds, suggesting that kidney development initiated at the same time in *Ntn1* conditional knockouts (Fig. 2A). Analysis of nephron progenitor niches at E15.5 and P0 revealed no obvious differences in their organization, suggesting normal progression of nephrogenesis (Fig. 2B; Fig. S2A). In addition, nephrogenesis qualitatively appeared to be normal in *Foxd1^GC/+^;Ntn1^fl/fl^* P0 kidneys as assessed by Lhx1 and Wt1 immunostaining and their respective labeling of the differentiating nephron segments (Lhx1-distal, Wt1-proximal/podocyte; Fig. S2B). Strikingly though, at P4, *Foxd1^GC/+^;Ntn1^fl/fl^* kidneys retained small clusters of Six2^+^ cells, whereas controls had exhausted their Six2^+^ population as expected (Fig. 2C). However, the Six2^+^ cells maintained differentiation capacity and by P5 were nearly exhausted (Fig. 2D).

In developing *Foxd1^GC/+^;Ntn1^fl/fl^* kidneys, the loss of *Ntn1* could affect the number of progenitor cells in nephron progenitor or ureteric tip niches, leading to hypoplasia. To assess cell numbers, we analyzed kidney sections at multiple timepoints and counted Six2^+^ nephron progenitors and E-cadherin (Ecad)^+^ ureteric tip cells within the niche (Fig. 2E,F). Apart from a transient difference in tip cell numbers at E13.5, neither population showed any significant differences between *Foxd1^GC/+^;Ntn1^fl/fl^* kidneys and controls when assessed at E13.5, E15.5 and P0 (Fig. 2F). Using the same method, we investigated whether the proliferative capacity of niche progenitors was impacted by measuring EdU incorporation into these populations as a measure of cell cycling (Fig. 2E,G). We found no significant differences in the number of EdU^+^ cells in either Six2^+^ or Ecad^+^ progenitor populations, suggesting that abnormal cell proliferation, at least at the stages analyzed, was not leading to hypoplastic kidneys (Fig. 2G).

### Deletion of *Unc5c* does not recapitulate the *Foxd1^GC/+^;Ntn1^fl/fl^* hypoplastic phenotype

**Fig. 3.**
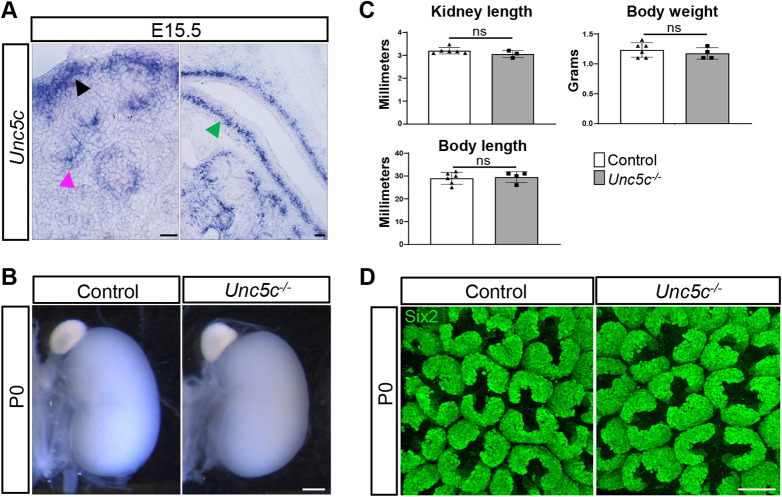
**Deletion of *Unc5c* does not recapitulate the *Foxd1^GC/+^;Ntn1^fl/fl^* hypoplastic phenotype.** (A) *In situ* hybridization showing expression of Ntn1 receptor *Unc5c* in nephron progenitors (black arrowhead), podocytes (magenta arrowhead) and the ureter smooth muscle layer (green arrowhead) at E15.5. (B) P0 images of whole kidneys show no significant difference in kidney size between *Unc5c*^−/−^ kidneys and controls. (C) Kidney length, body length and body weight were measured at P0 and showed no significant difference. Graphs show mean±s.d. with points representing biological replicates. Unpaired, two-tailed Student's *t*-test. ns, not significant. (D) Wholemount immunostaining and surface views show normal organization of nephron progenitor niches in *Unc5c^−/−^* kidneys at P0 (Six2, green). Scale bars: 50 µm (A); 500 µm (B); 40 µm (D).

Our previous nephron progenitor RNA-seq data, as well as recent scRNA-seq data, indicate that the netrin receptor *Unc5c* is expressed by nephron progenitors (Combes et al., 2019; O'Brien et al., 2018, 2016). *In situ* hybridization of *Unc5c* confirmed expression by the nephron progenitors, as well as podocytes and smooth muscle cells of the ureter (Fig. 3A). Owing to the nephron progenitor expression, we wondered whether disrupted signaling via Ntn1 to the *Unc5c*^+^ nephron progenitors might account for the smaller kidney size and delay in cessation of nephrogenesis. We assessed the kidney phenotype of *Unc5c*^−/−^ mice (Ackerman et al., 1997) and found no significant difference in kidney size compared with controls, in addition to no significant differences in animal body length and weight (Fig. 3B,C). The Six2^+^ nephron progenitor niches appear to be normal, and no other overt phenotypes were observed at any stages examined up to birth (Fig. 3D). The *Unc5c*^−/−^ pups die within 24-48 h of birth when maintained on the C57Bl6/J background, most likely due to neurological defects. Therefore, this precluded the analysis of P4 kidneys to determine whether nephrogenesis was extended. However, with the lack of any significant hypoplastic kidney phenotype in the *Unc5c*^−/−^ mice, this suggests that Ntn1 signaling through Unc5c is not a major regulator of kidney development or that other receptors may functionally compensate.

### Ntn1 mediates vascular patterning in the developing kidney

**Fig. 4.**
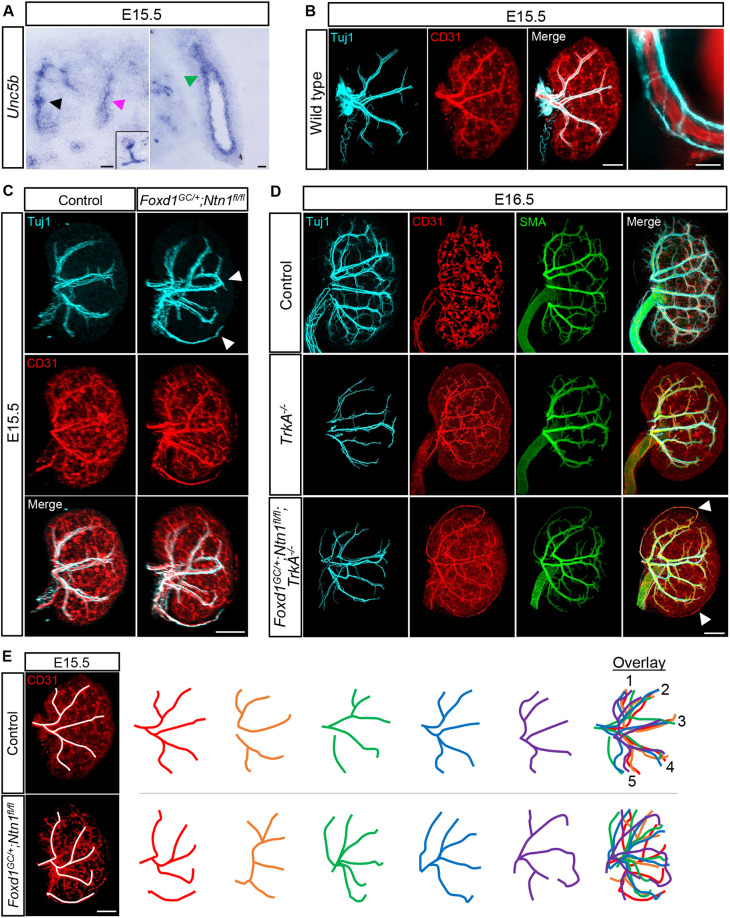
***Foxd1^GC/+^;Ntn1^fl/fl^* kidneys display aberrant neurovascular patterning.** (A) *In situ* hybridization at E15.5 shows the expression of Ntn1 receptor *Unc5b* in kidney endothelium (black arrowhead and inset of glomerular endothelium), the secondary branches/generations of the collecting duct (magenta arrowhead) and ureter (green arrowhead). (B) E15.5 wholemount immunostain of a wild-type kidney shows tracking of Tuj1^+^ (cyan) axonal projections with the vasculature (CD31, red). Far right panel shows an independent magnified image of a neurovascular bundle. (C) Wholemount immunostains show the organization of the nerves and vasculature at E15.5 in *Foxd1^GC/+^;Ntn1^fl/fl^* kidneys and controls. The nerves (Tuj1, cyan) track with the endothelium (CD31, red) in all genotypes. White arrowheads highlight the abnormal patterning observed in *Foxd1^GC/+^;Ntn1^fl/fl^* kidneys such as looping and ectopic neurovascular bundles. (D) E16.5 wholemount immunostains of control, *TrkA^−/−^* and *Foxd1^GC/+^;Ntn1^fl/fl^;TrkA^−/−^* kidneys show the organization of nerves (Tuj1, cyan), endothelium (CD31, red) and smooth muscle cells (SMA, green). Innervation of the kidney is reduced in *TrkA* knockouts, but abnormal patterning persists in *Foxd1^GC/+^;Ntn1^fl/fl^;TrkA^−/−^* kidneys, with vasculature not associated with axons (arrowheads) still showing abnormal patterning. (E) Depiction of tracing (white lines) of the CD31^+^ major vascular branches (left panel). Tracing of five control kidneys (top panel, colored lines) shows predictable patterns of five main lateral arterial branches which can be overlayed. *Foxd1^GC/+^;Ntn1^fl/fl^* kidneys (bottom panel, colored lines) show stochastic patterning with the absence of any predictable patterning. Scale bars: 50 µm (A, B magnification); 300 µm (B merge, C-E).

Both axons and endothelium respond to guidance cues such as Ntn1 using specific receptors. Unc5b has been shown to mediate angiogenesis (Larrivée et al., 2007; Lu et al., 2004) and *in situ* hybridization at E15.5 shows that *Unc5b* is expressed by the kidney endothelium as well as the ureteric epithelium, although is excluded from the most peripheral ureteric branches (Lindström et al., 2018) (Fig. 4A). During kidney development, axonal projections tightly associate and track with the vasculature, forming tight neurovascular bundles (Fig. 4B). Therefore, we included both nerves and vessels in our analyses, as both have the potential to respond to Ntn1 guidance through their respective receptors. To assess overall organization and patterning of these networks, we used wholemount immunostaining in combination with light-sheet microscopy to generate 3D visualizations. Control kidneys show the formation of an extensive CD31^+^ (Pecam1^+^) endothelial network by E15.5 (Fig. 4C). Tuj1^+^ (Tubb3) axons track closely with the main arterial branches at this stage, which form a pattern consistent with those observed by Daniel et al. (2018) (Fig. 4C; Movie 1). In contrast, we observed aberrant neuronal and vascular patterning in the *Foxd1^GC/+^;Ntn1^fl/fl^* kidneys (Fig. 4C; Movie 2). The example shown features looping of the arterial tree within the kidney cortex and ectopic vessels on the kidney surface (Fig. 4C, arrowheads), supporting a role for Ntn1 in mediating proper neurovascular patterning.

As both axons and endothelium can respond to Ntn1 guidance cues, either may be mediating the neurovascular patterning. Therefore, we sought to differentiate which one is the primary responder to stromal-derived Ntn1 signals. Deletion of the *TrkA* receptor for nerve growth factor (NGF) induces apoptosis of peripheral neurons such as those that innervate the kidney (Smeyne et al., 1994). We used the *TrkA* (*Ntrk1*) knockout (Liebl et al., 2000) to investigate whether kidney vascular patterning is disrupted in the setting of reduced kidney innervation. In *TrkA* knockout kidneys, the endothelial (CD31) and arterial (α-smooth muscle actin, SMA; Acta2) patterns were qualitatively normal at E16.5 (Fig. 4D). However, when combined with the *Foxd1^GC/+^;Ntn1^fl/fl^* alleles to generate *Foxd1^GC/+^;Ntn1^fl/fl^;TrkA^−/−^* animals, the kidney endothelium still displayed abnormal patterning similar to *Foxd1^GC/+^;Ntn1^fl/fl^*, including looping and ectopic vessels (Fig. 4D). The ectopic and looping vessels were not associated with Tuj1^+^ axons (Fig. 4D, arrowheads), suggesting that the endothelium is the primary responder to Ntn1 guidance cues, with nerves following the SMA^+^ vascular pattern as it is established.

Tracing of the main CD31^+^ arterial branches shows that controls form a predictable pattern, as expected, which can be overlayed into five main branches from a lateral view (Daniel et al., 2018) (Fig. 4E). However, the *Foxd1^GC/+^;Ntn1^fl/fl^* kidneys have a breakdown of predictable patterning and overall stochastic patterning (Fig. 4E). Though not a focus of this study, the cortical vasculature around the developing nephron progenitor niches of *Foxd1^GC/+^;Ntn1^fl/fl^* kidneys is also disrupted, featuring disorganized and ectopic vasculature (Fig. S3A). Notably, the presence of Ter119^+^ (Ly76^+^) erythroid cells indicates that the blood vessels are perfused in both control and *Foxd1^GC/+^;Ntn1^fl/fl^* kidneys at E15.5, although contribution from hemo-vasculogenesis is also possible (Sequeira Lopez et al., 2003) (Fig. S3B).

Netrin 3 (*Ntn3*) is a functionally related guidance cue that can engage Ntn1 receptors (Wang et al., 1999). It is also expressed, albeit at lower levels, by the stromal progenitors, which we confirmed by *in situ* hybridization (Combes et al., 2019; England et al., 2020) (Fig. S4A). To determine whether Ntn3 also mediated vascular patterning in the developing kidney, we generated a *Ntn3* knockout line using CRISPR/Cas9 to delete the majority of the *Ntn3* coding region (see Materials and Methods). However, we did not observe any neurovascular patterning defects or differences in kidney size in *Ntn3* knockouts or any exacerbation of the *Foxd1^GC/+^;Ntn1^fl/fl^* phenotype when double knockouts (*Foxd1^GC/+^;Ntn1^fl/fl^;Ntn3^−/−^)* were generated (Fig. S4B,C). We also investigated whether *Unc5c* knockout kidneys displayed altered patterning, even though they were not hypoplastic. The neurovasculature appeared to be largely normal with no ectopic vessels, aberrant looping or qualitative reductions (Fig. S4D). Altogether, these data suggest Ntn1 is the primary netrin guidance cue responsible for kidney vascular patterning acting through receptors such as Unc5b on the endothelium.

### Vascular smooth muscle cell coverage is reduced in *Foxd1^GC/+^;Ntn1^fl/fl^* kidneys

**Fig. 5.**
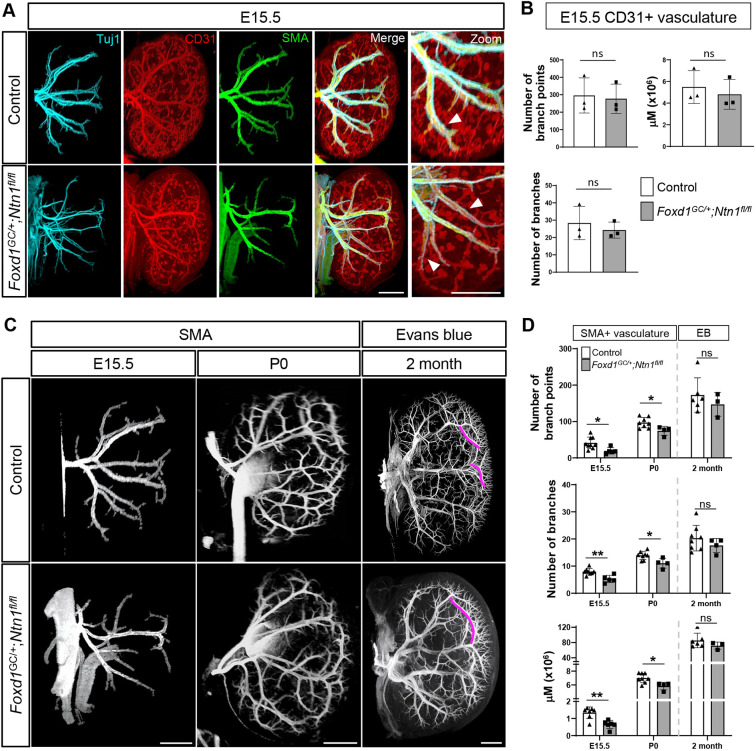
***Foxd1^GC/+^;Ntn1^fl/fl^* kidneys have normal endothelial metrics but reduced smooth muscle coverage.** (A) E15.5 wholemount immunostaining of smooth muscle cells (SMA, green), axons (Tuj1, cyan) and endothelium (CD31, red) shows a qualitative delay in smooth muscle cell association with the endothelium (white arrowheads, zoom) suggesting a potential delay in vascular maturation. (B) Quantitation of CD31^+^ endothelial metrics including the number of branches, branch points and total endothelium (total additive length, µm) at E15.5 shows no statistical differences between controls and *Foxd1^GC/+^;Ntn1^fl/fl^* kidneys. (C) Wholemount immunostains of E15.5 and P0 kidneys with SMA (white) showing a qualitative reduction of vascular coverage in *Foxd1^GC/+^;Ntn1^fl/fl^* kidneys. The injection of Evans Blue dye at 2 months to label adult kidney vasculature shows a well-developed arterial tree with some remnants of abnormal patterning such as elongated branches in *Foxd1^GC/+^;Ntn1^fl/fl^* kidneys. Magenta lines abut normal (top) and abnormal (bottom) branches. (D) Quantitation of SMA^+^ arterial metrics including the number of branches, branch points and total vasculature length (µm) at E15.5 and P0 shows a deficit in smooth muscle metrics of *Foxd1^GC/+^;Ntn1^fl/fl^* kidneys. Quantitation of Evans Blue-labeled vasculature at 2 months reveals that *Foxd1^GC/+^;Ntn1^fl/fl^* kidney metrics are similar to controls. Graphs show mean±s.d. with points representing biological replicates. *P≤0.05, **P≤0.01 (unpaired, two-tailed Student's *t*-test). ns, not significant. Dotted demarcation between P0 and 2 months (D) denotes the differences in labeling, with early stages labeled by SMA and 2 months perfused with Evans Blue. Scale bars: 300 µm (A, C E15.5); 500 µm (C P0); 1000 µm (C 2 month).

As the endothelium matures it is covered by a layer of vSMCs. These cells provide support, maintain arterial wall integrity and can direct innervation through the release of guidance molecules (Brunet et al., 2014; Potente and Mäkinen, 2017). To assess vascular maturity and determine whether vSMCs were impacted in *Foxd1^GC/+^;Ntn1^fl/fl^* animals, we investigated SMA coverage of the endothelium in wholemount immunostained kidneys. Qualitatively, we observed a reduction in SMA^+^ signal associated with the endothelium at E15.5 in *Foxd1^GC/+^;Ntn1^fl/fl^* kidneys (Fig. 5A; Movies 3,4). Specifically, there was an increasing reduction in SMA coverage of CD31^+^ vessels as they extended to the cortex (Fig. 5A, arrowheads). To quantitatively assess vascular metrics, we used Imaris imaging and analysis software to generate wireframes of the vascular trees which were then used to measure vascular parameters such as length and branching (Fig. S5A). Quantification of the CD31^+^ endothelium in *Foxd1^GC/+^;Ntn1^fl/fl^* kidneys revealed that branching metrics and total vessel length were not significantly affected at E15.5 (Fig. 5B). In contrast, quantification of the SMA^+^ arterial vasculature showed reductions at E15.5 and P0 in *Foxd1^GC/+^;Ntn1^fl/fl^* kidneys (Fig. 5C,D). On average, E15.5 kidneys from *Foxd1^GC/+^;Ntn1^fl/fl^* animals have greater than a 20% reduction in SMA^+^ arterial metrics including branch number, branch points, and the total length of the vSMC-lined vasculature (Fig. 5D)**.** At P0, reductions in these three vascular parameters were still significant highlighting the persistence of this phenotype throughout development (Fig. 5D). These data suggest that Ntn1 loss impacts proper smooth muscle coverage of the endothelium during kidney development.

To determine whether the defects observed in development extended into adulthood, we labelled the vasculature of 2- to 3-month-old *Foxd1^GC/+^;Ntn1^fl/fl^* animals by perfusing Evans Blue (Fig. 5C). Evans Blue is a bis-azo dye that fluoresces only while bound to albumin and labels the perfused renal vasculature, specifically the arterial network (Honeycutt and O'Brien, 2021). We used this methodology due to the difficulties with efficiently immunolabeling vascular networks in whole adult kidneys and rationalized that perfused vessels would be covered by mural cells such as vSMCs. At 2 months, quantification of *Foxd1^GC/+^;Ntn1^fl/fl^* kidneys labeled with Evans Blue revealed no significant differences in vascular metrics, although all three metrics trended towards a reduction (Fig. 5D). Tissue sections show the presence of qualitatively equivalent endothelial networks and SMA^+^ arteries between controls and *Foxd1^GC/+^;Ntn1^fl/fl^* kidneys at 3 months (Fig. S5C). At both 2 and 3 months of age, ectopic vessels were no longer observed and the kidney vasculature had fewer visible defects, although this is difficult to ascertain owing to the highly complex vascular network that has formed. However, differences were noted such as meandering, elongated vessels near the cortex (Fig. 5C, magenta lines; Fig. S5B, red dotted lines) and areas of reduced branching (Fig. S5B, yellow arrows). In addition, we occasionally observed clouds of Evans Blue signal outside the vascular network (Fig. S5B, white arrowhead), indicating possible leakage and compromise to vessel integrity.

### RNA-seq reveals differences in an array of cellular programs of *Foxd1^GC/+^;Ntn1^fl/fl^* kidneys

With the alterations to vascular patterning, maturity and proper kidney development in *Foxd1^GC/+^;Ntn1^fl/fl^* animals, we rationalized that interrogating transcriptional programs could provide insights into the molecular underpinnings of the phenotypes. To this end, we performed bulk RNA-seq on whole kidneys at E15.5, the developmental stage at which we observed significant vascular and hypoplastic phenotypes. To account for any exacerbated transcriptional differences due to the loss of one *Foxd1* allele from the *GFP-Cre* insertion, we compared *Foxd1^GC/+^;Ntn1^fl/fl^* homozygous kidneys with *Foxd1^GC/+^;Ntn1^fl/+^* heterozygous kidneys (Table S1). We used a 1.2-fold cutoff, as large fold changes may be obscured due to the bulk tissue analysis. Gene ontology analyses were then performed on up- and downregulated genes with a ≥1.2-fold change and a *P*≤0.05 to identify biological processes enriched in either category (Tables S2 and S3).

**Fig. 6.**
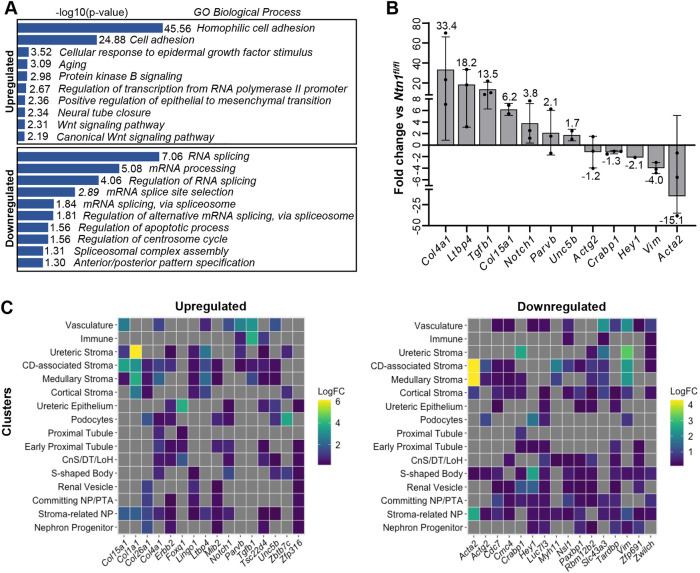
**Gene expression analyses of E15.5 kidneys highlight processes and cell types affected by Ntn1 loss.** (A) Gene ontology (GO) analysis of genes upregulated (top) or downregulated (bottom) in *Foxd1^GC/+^;Ntn1^fl/fl^* (homozygous) kidneys compared with *Foxd1^GC/+^;Ntn1^fl/+^* (heterozygous) controls. RNA-seq was performed on E15.5 kidneys (three biological replicates/genotype) to assess changes in gene expression. Shown are GO biological processes enriched in both categories. Genes were considered up- or downregulated with a ≥1.2-fold-change, *P*≤0.05. (B) Up- and downregulated genes with established roles in the kidney and/or vasculature were analyzed by qPCR to confirm their relative up- or downregulation in *Foxd1^GC/+^;Ntn1^fl/fl^* E15.5 kidneys. *Ntn1^fl/fl^* kidneys were used as controls. All genes followed the same trends as the RNA-seq. Graphs show mean±s.d., three biological replicates. Missing replicate, no Ct value. (C) The top 10 genes up- or downregulated in addition genes tested by qPCR were mapped against scRNA-seq data (Combes et al., 2019) to assess their normal relative enrichment in the different kidney cell types (clusters) at E18.5. Upregulated genes are enriched in the stromal cell clusters and vasculature. Downregulated genes are similarly enriched in stromal clusters and vasculature as well as developing nephron clusters. CD, collecting duct; CnS, connecting segment; DT, distal tubule; LogFC, log fold change; LoH, loop of Henle; NP, nephron progenitor; PTA, pretubular aggregate.

Genes upregulated in *Foxd1^GC/+^;Ntn1^fl/fl^* kidneys were associated with biological processes including cell adhesion, Wnt signaling, cell migration and a variety of developmental processes (heart, nervous system, lung) (Fig. 6A; Table S2). Top genes that were upregulated (*P*<0.01) included *Col26a1*, *Col1a1* and *Tgfb1*. Although there are well-known links between TGF-β1 and collagen production in fibrosis, less is known about the relationship during tissue development (Kim et al., 2018). However, TGF-β1 has well-established roles in smooth muscle cell differentiation and, interestingly, latent transforming growth factor-β-binding protein-4 (*Ltbp4*) was also significantly upregulated (1.46-fold, *P*=0.04) (Guo and Chen, 2012). It was recently uncovered that Ltbp4 stimulates angiogenesis *in vitro* as well as in the mature kidney (Su et al., 2021). Although only showing weak significance (0.05<*P*<0.1), other angiogenic factors such as *Unc5b*, *Notch1* and *Parvb* were upregulated (Javerzat et al., 2009; Larrivée et al., 2007; Limbourg et al., 2005; Lu et al., 2004; Pitter et al., 2018) (Table S2). We validated the upregulation of a subset of the genes by qPCR and confirmed that *Tgfb1*, *Ltbp4*, *Notch1*, *Unc5b*, *Parvb* and additional collagens *Col4a1* and *Col15a1* all showed general upregulation compared with *Nnt1^fl/fl^* controls (Fig. 6B). Together, these changes in gene expression correlate with the phenotypes we observed and suggest that the loss of Ntn1 signaling likely affects processes including angiogenesis and vSMC maturation/adhesion/migration, the latter of which (vSMC migration) was validated by Luo et al. (2023) in their complementary studies.

As mentioned previously, biological processes associated with Wnt signaling were also upregulated. Wnt signaling is a major signaling pathway involved in kidney development, modulating processes such as progenitor proliferation and differentiation (Wang et al., 2018). Genes upregulated included *Foxq1*, *Tle3*, *Tcf7l1*, *Tcf7*, *Axin1* and *Axin2*, genes all largely associated with the canonical Wnt pathway (Christensen et al., 2013; Clevers, 2006; Liu et al., 2022). Although we did not observe significant alterations to progenitor proliferation (Fig. 2G), nephron progenitor differentiation could be affected, and Luo et al. (2023) show that glomerular numbers, a proxy for the number of nephrons formed, are reduced in *Foxd1^GC/+^;Ntn1^fl/fl^* mature kidneys.

Genes downregulated in *Foxd1^GC/+^;Ntn1^fl/fl^* kidneys were significantly associated with processes related to RNA splicing. Intriguingly, recent studies have investigated splicing in the developing mouse kidney and uncovered splice isoform switching in the early differentiating nephrons, in addition to a role for splice regulators in modulating ureteric branching and nephron differentiation (Bebee et al., 2016; Wineberg et al., 2020). Thus, RNA splicing defects may be related to the hypoplastic phenotype. Genes expressed by and important for smooth muscle cell differentiation and function including *Acta2* (αSMA,) *Actg2* (γSMA), *Vim* and *Myh11* were also downregulated, supporting the reduced SMA^+^ coverage we observed in *Foxd1^GC/+^;Ntn1^fl/fl^* kidneys (Table S3; Fig. 5) (Chi et al., 2008; Morano et al., 2000; Sorokin et al., 2020; van Engeland et al., 2019; Yap et al., 2021). We confirmed the downregulation of *Acta2*, *Actg2* and *Vim* in *Foxd1^GC/+^;Ntn1^fl/fl^* kidneys compared with *Ntn1^fl/fl^* controls by qPCR. In addition, we confirmed *Crabp1* and *Hey1* downregulation by qPCR as retinoic acid signaling and Notch signaling, respectively, play important roles in kidney development and may be related to the hypoplastic phenotype (Mukherjee et al., 2019; Rosselot et al., 2010).

Although genes with known roles in kidney and vascular development were identified in our RNA-seq comparisons, many of the genes have unknown functions in these processes. Understanding what cell types normally express these genes can provide insights into complex phenotypes such as those of our *Ntn1* conditional knockouts. Therefore, we took the top ten genes from our up- and downregulated gene lists that were sorted by significance (*P*-value), in addition to the genes tested by qPCR and which are known cellular markers, and assessed their normal expression in cells of the developing kidney. To do this, we used publicly available scRNA-seq data from the developing wild-type E18.5 kidney and plotted the fold change enrichment of our genes of interest in all the cell types (clusters) identified in the study (Combes et al., 2019). Heatmaps representing the log fold change in each cluster were generated for the up- and downregulated genes independently (Fig. 6C).

**Fig. 7.**
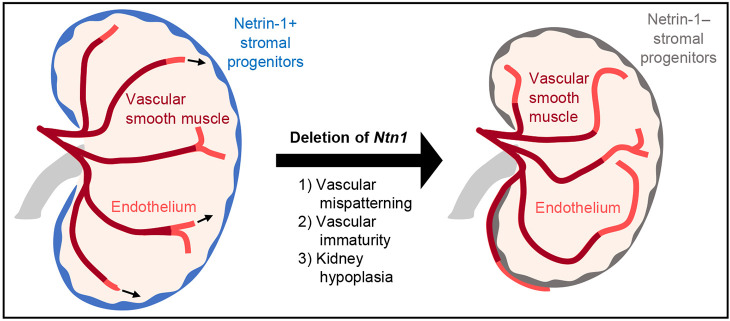
**Ntn1 guides vascular patterning and maturity in the developing kidney.** Schematic for the role of Ntn1 in guiding vascular patterning and maturation during development. Ntn1 is secreted by stromal progenitors (blue) in the developing kidney. When *Ntn1* is deleted (arrow), the vasculature is mispatterned and there is reduced vascular smooth muscle cell (dark red) association with the endothelium (light red). Additionally, the kidneys are hypoplastic (right) with an extended nephrogenic period and altered gene expression.

Upregulated and downregulated genes displayed a similar trend, with many of the genes being enriched in the stromal clusters and vasculature (endothelium). This includes the ‘Stroma-related nephron progenitor (NP)’ cluster that was reported by Combes et al. to display signatures of both stromal and nephron progenitors and represent a potential sequencing artifact (Combes et al., 2019). Interestingly, this cluster also displays signatures of smooth muscle with the expression of *Acta2*, *Actg2* and *Myh11* and thus the overall enrichment of up- and downregulated genes in this cluster indicates potential alterations to smooth muscle programs. The downregulated genes show additional enrichment in cell clusters that represent differentiating nephrons including ‘Committing nephron progenitor (NP)/pretubular aggregate (PTA)’, ‘Renal vesicle’ and ‘S-shaped body’. Effects on the programs of differentiating nephrons can impair their proper development and relate to the hypoplastic phenotype we and Luo et al. observed and the glomerular deficiencies identified in Luo et al. (2023). Altogether, these analyses suggest that these cell types – stroma, endothelium, smooth muscle and differentiating nephrons – are the ones most affected by the loss of Ntn1, which correlates with the vascular patterning/maturity and hypoplastic phenotypes of *Foxd1^GC/+^;Ntn1^fl/fl^* kidneys (Fig. 7).

## DISCUSSION

In this study, we demonstrate that stromal progenitor *Ntn1* is necessary for proper patterning and maturation of the kidney arterial network. *Foxd1^GC/+^;Ntn1^fl/fl^* kidneys have visible and quantifiable stochastic vascular patterning defects, with reductions in most arterial metrics during embryogenesis and postnatally. These differences in vascular metrics largely resolve in adulthood, although patterning defects are still observed. Furthermore, deletion of *Ntn1* resulted in a delay in the cessation of nephrogenesis and kidney hypoplasia. Altogether, our studies and those in the study by Luo et al. (2023), which uncover similar and complementary findings, highlight the significance of Ntn1 to proper kidney vascularization and associated development.

### Sorting out the vascular phenotypes: the role of Ntn1 in patterning and maturity

Although we find differences in vascularization in the absence of Ntn1, our analyses relate specifically to the larger arteries and arterioles of the vascular tree. SMA will only label vasculature with sufficient smooth muscle coverage, typically larger arteries and arterioles, missing the capillaries which are primarily covered by NG2^+^ pericytes. However, Luo et al. (2023) demonstrate a lack of proper NG2^+^ pericyte localization in early development, suggesting that Ntn1 is an important signaling molecule for all vascular mural cells. In addition, we did not investigate venous formation and patterning during development, primarily owing to a lack of appropriate and agreed-upon markers of the venous vasculature (Daniel et al., 2018). Veins are also covered with significantly fewer SMCs due to a decreased need to regulate pressure and tone. This makes SMA an unreliable venous marker at lower magnifications, such as those used by light-sheet microscopy to image the vasculature. Therefore, additional studies are necessary to determine to what extent the entire vascular plexus is disrupted in *Foxd1^GC/+^;Ntn1^fl/fl^* kidneys.

Vascular patterning and maturity are both disrupted in the *Foxd1^GC/+^;Ntn1^fl/fl^* kidneys in our study. Luo et al. (2023) further define the maturity phenotype as being associated with a decrease in *Klf4* expression and accumulation of mural cells on the surface of the kidneys. We did not observe any obvious accumulation of mural cells at E15.5 or later stages (Fig. 5). This may be due to the ectopic accumulation resolving in our mice by E15.5, the earliest stage at which we assess SMA localization. Alternatively, our studies use different imaging modalities to assess the vascular networks. The confocal imaging used by Luo et al. (2023) provides higher resolution than our light-sheet, and may better capture the surface accumulation of SMA^+^ cells. Whether Klf4 is associated with the phenotype in our studies is unknown. We did not find a difference in *Klf4* expression in our E15.5 RNA-seq (Table S1), although this is 2 days later than the RNA-seq performed by Luo et al. (2023) and *Klf4* levels may have normalized by this time or differences are masked by the normal increase of *Klf4* in other kidney cell types. Therefore, the observed maturation phenotypes may initiate at the earliest stages of arterialization, around E13.5, and before our analysis at E15.5. Our RNA-seq analyses point to additional players that may be downstream of initiating events, including altered TGF-β1 signaling and collagen deposition (Fig. 6), which could further influence or support the vascular phenotypes. The timing of phenotype onset and how it is perpetuated could explain the differences between our study and that of Luo et al. (2023), highlighting the complementary nature of our analyses.

VSMCs *in vitro* express netrin receptors such as neogenin (*Neo1*) and Unc5b and signaling through these receptors supports endothelial vSMC coverage (Lejmi et al., 2014, 2008). As such, deletion of *Ntn1* from the stroma may ultimately impact vSMC maturation or recruitment in the kidney and, in turn, affect vascular patterning. This is similar to the findings of Hurtado et al., who showed that Pbx1 mediates mural cell maturation, ultimately influencing proper vascular patterning within the developing kidney (Hurtado et al., 2015). Alternatively, patterning of the endothelium may influence vascular maturation. Deletion of *Unc5b* from the kidney endothelium and the assessment of vascular patterning and maturity would potentially help sort out if the patterning defects we observed are independent of vSMC coverage. The lack of significant phenotypes in the *Unc5c* knockouts suggest it is either not a major receptor for Ntn1 in the kidney or it is functionally redundant (Fig. 3; Fig. S4D). Considering Ntn1 can signal through a large number of receptors, including integrins, and displays receptor independent cross-repressive activities with LRIG proteins, uncovering the receptor(s) and downstream signaling mechanisms of Ntn1 in the kidney may prove challenging (Abdullah et al., 2021; Abraira et al., 2008; Stanco et al., 2009; Sun et al., 2011; Yebra et al., 2003).

### Potential mechanisms behind *Foxd1^GC/+^;Ntn1^fl/fl^* hypoplasia

In regard to the reduced kidney size of *Foxd1^GC/+^;Ntn1^fl/fl^* animals, the explanation for this phenotype is less clear. However, our RNA-seq analyses provide insights into putative mechanisms. Canonical Wnt signaling-associated genes *Tle3*, *Tcf7l1*, *Tcf7*, *Axin1* and *Axin2* were upregulated and Wnt signaling has well-established roles in kidney development (Wang et al., 2018). Recently, it was shown that Ntn1 enhances the interaction of Unc5b with LRP6, a Wnt co-receptor, thereby activating Wnt/β-catenin signaling and linking the two signaling pathways in blood-brain barrier integrity (Boyé et al., 2022). However, with Wnt pathway genes upregulated in the absence of Ntn1, this cooperative signaling may not be active in kidney endothelium. The upregulation of Wnt pathway genes is more likely to represent alterations to nephron differentiation where canonical Wnt signaling is crucial (Carroll et al., 2005; Karner et al., 2011). Recent studies have also shown that stromal β-catenin activation affects proper kidney development (Boivin et al., 2015; Drake et al., 2020). Assessing individual cell types for upregulated Wnt signaling and quantitative analysis of nephron structures would be necessary to sort out the responsible cell type(s).

The kidney hypoplasia observed in our *Foxd1^GC/+^;Ntn1^fl/fl^* animals may be linked to the relative vascular immaturity we observed. This could perturb metabolic processes and ultimately affect proper kidney growth and prolong the nephrogenic period (Liu et al., 2017). However, we did not observe significant changes to metabolic processes in our RNA-seq data; instead we saw disruptions to genes associated with alternative splicing (Fig. 6A; Table S3). With recent reports suggesting a role for RNA splicing in proper kidney development, these changes in gene expression may be tied to the hypoplastic phenotype (Bebee et al., 2016; Wineberg et al., 2020). In addition, genes associated with kinetochore assembly and function (*Zwilch* and *Nsl1*) and the cell cycle (*Cdc7*) were amongst the top ten downregulated genes (Fig. 6C; Table S3) (Kline et al., 2006; Lei et al., 1997; Suski et al., 2022; Williams et al., 2003). These genes were enriched in the developing nephron clusters and may indicate improper cell division in the *Foxd1^GC/+^;Ntn1^fl/fl^* kidneys, thus affecting nephrogenesis. However, these genes are also enriched in the vascular endothelium (Fig. 6C). These findings highlight the complexity of our phenotypes and difficulty in sorting out mechanisms – whether loss of Ntn1 signals has primary or secondary (through the vascular defects) effects on kidney development. With the difficulty of culturing primary kidney cells *ex vivo* and the regression of vessels in kidney explants (Ryan et al., 2021), complex genetic crosses with the *Foxd1^GC/+^;Ntn1^fl/fl^* mice are likely necessary to start parsing out the phenotypes.

### Implications for physiology and kidney replacement strategies

Although the *Foxd1^GC/+^;Ntn1^fl/fl^* adults appear to have attained equivalent vascular metrics, the metrics we assess do not provide information on where the branching occurs or the length of any individual branch. Therefore, there may be patterning defects that are not identified in our quantitative metrics or that can be easily visualized in adults where the vascular network is much more complex. We do observe some longer arterial branches in the adult kidneys as well as reduced branching along arteries (Fig. 5C; Fig. S5B). Although our basic quantitative assessment shows that branch numbers, branch points and total length are roughly equivalent between controls and *Foxd1^GC/+^;Ntn1^fl/fl^* kidneys, it does not inform of different patterns that may still generate similar metrics. To resolve this, we will need to develop more refined metrics that can assess differences in vascular network spatial orientations.

Whether the development of a kidney with abnormal vascular patterns affects physiological function remains unknown. *Foxd1^GC/+^;Ntn1^fl/fl^* animals do live into adulthood, with no overt kidney or health phenotypes. If the smaller vascular beds in important functional regions of the nephron, such as the glomerulus and tubules, are organized properly, then physiological function may be largely unperturbed. However, injury, disease or aging may exacerbate or induce changes in physiological function in *Foxd1^GC/+^;Ntn1^fl/fl^* animals and will be the focus of future studies. Understanding whether precise vascular patterning is crucial to function, both in health and under physiological stress, will help inform efforts to vascularize tissues for functional transplant.

As the kidney lacks regenerative mechanisms, *de novo* kidney engineering and *in situ* repair using *in vitro*-generated nephrons or induced pluripotent stem cell-derived renal organoids are considered attractive therapeutic possibilities (Morizane et al., 2015; Taguchi et al., 2014; Takasato et al., 2015; Wu et al., 2018). Unfortunately, neither treatment is currently approaching clinical relevance (Bantounas et al., 2018). With current protocols, renal organoids form basic nephron structures but eventually become necrotic from a deficit of oxygen and nutrients as functional endothelium is not present (Grebenyuk and Ranga, 2019). Interestingly, cells expressing the pan-endothelial marker CD31 occur *de novo* in organoids, but without perfusion they do not form connected, functional networks, resulting in a lack of proper lumenization, flow and patterning, and their eventual regression (Homan et al., 2019; Ryan et al., 2021; Taguchi et al., 2014; Wu et al., 2018). In addition, the vessels would need to obtain appropriate mural cell coverage to support function. Therefore, studies such as ours and those of Luo et al. (2023) provide crucial insights into how the renal vasculature is patterned, matures and contributes to kidney development, and will help guide kidney engineering strategies.

## MATERIALS AND METHODS

### Animals

All animal studies were approved by the Office of Animal Care and Use at the University of North Carolina (UNC) at Chapel Hill and the UNC Institutional Animal Care and Use Committee (IACUC). Procedures were performed under IACUC-approved protocols for mice (16-276.0, 19-183.0, 22-136). Animal husbandry was performed by the UNC Division of Comparative Medicine (DCM). All animals in this study were kept in environmental conditions with temperatures of 68-74°F and humidity ranges of 30-70%. No more than five adult animals were housed per cage, and all cages were provided a 12 h light/12 h dark cycle. Animals were fed LabDiet PicoLab Select Rodent 50 IF/6F (5V5R). Timed matings were set up and plugs were ascertained by visual and probed inspection. The presence of a plug was considered 0.5 days gestation. The day of birth (E19.5) was considered P0, pups born before or after E19.5 were not used in these studies.

All mouse lines were maintained on the C57Bl6/J background (The Jackson Laboratory, strain #000664). Details of genetically edited mouse lines used are as follows: *Foxd1^GC/+^* (strain #012463) (Humphreys et al., 2010; Kobayashi et al., 2014) and *Ntn1^fl/fl^* (strain #028038) (Bin et al., 2015) mice were purchased from The Jackson Laboratory, *TrkA* knockout mice (Liebl et al., 2000) were a gift from Lino Tessarollo (National Institutes of Health National Cancer Institute Center for Cancer Research, USA) and *Unc5c* knockout mice (Ackerman et al., 1997) were a gift from Susan Ackerman (University of California, CA, USA). *Foxd1^GC/+^;Ntn1^fl/fl^* mice were generated by crossing Cre-expressing *Foxd1^GC/+^;Ntn1^fl/+^* to *Ntn1^fl/fl^* mice unless otherwise noted. Controls were either *Ntn1^fl/fl^* or *Foxd1^GC/+^;Ntn1^fl/+^* as we did not find any qualitative or quantitative differences between genotypes for any of the analyses. Genotyping primers are listed in Table S4.

*Ntn3* knockout mice were generated by the UNC Animal Models Core using CRISPR/Cas9-mediated genome editing. Guide RNAs *Ntn3*-gRNA1 (CGAGGAATCGCAGACGCGAT, exon 1, chr17:24208777-24208796) and *Ntn3*-gRNA2 (GAAAGCGTGTCCACGAGCCG, intron 5, chr17:24206891-24206910) were designed to delete the majority of the *Ntn3* coding region spanning exons 1-6. Guide RNAs were generated within our lab and provided to the animal models core at estimated concentrations of 500 ng/µl and 450 ng/µl, respectively. Guide RNAs were prepared as in O'Brien et al. (2018). Recombinant Cas9 protein was expressed and purified by the UNC Protein Expression Core using a plasmid provided by the Animal Models Core. A harvest of 210 fertilized embryos (zygotes) was obtained from nine super-ovulated *Ntn1^fl/fl^* females crossed to *Foxd1^GC/+^;Ntn1^fl/fl^* males. Zygotes were electroporated with two different reagent concentrations diluted in OptiMEM medium (Gibco) and then implanted in pseudo-pregnant recipient females. Mix 1: 1.2 µM Cas9 protein, 47 ng/µl each guide RNA; 102 embryos electroporated, 85 embryos implanted in four B6D2F1 recipients. Mix 2: 400 nM Cas9 protein, 25 ng/µl each guide RNA; 108 embryos electroporated, 95 embryos implanted in four B6D2F1 recipients. Of the resulting pups, one male containing the correct deletion as confirmed by Sanger sequencing over the deletion site was used as the founder. Brain and kidney tissue was harvested and qPCR performed to confirm the loss of *Ntn3* transcripts in knockout animals. *Ntn3* knockout animals are available upon request. Genotyping primers are listed in Table S4.

A mix of male and female embryos, pups, and adults were used for all studies. Although sex was noted, we did not find any statistical differences and have therefore combined sexes for all experiments. We performed a minimum of three biological replicates for all studies.

### Kidney and body size measurements

Kidney length measurements were performed using FIJI software to analyze images taken at 2× magnification on a Leica dissecting scope. Standardization and calibration of FIJI measurements were performed using a 1 µm-1 mm micrometer taken at the same magnification. Body length was measured similarly from head to tail. Body weight was obtained by placing the pup on a standard lab scale.

### *In situ* hybridization

*In situ* hybridizations were performed on wild-type cryosectioned E15.5 and P2 Swiss Webster (SW, Taconic) kidneys using the ‘Section *In Situ* Hybridization (SISH) on kidney sections’ protocol (Krautzberger and Guo; McMahon Lab; https://www.gudmap.org/chaise/record/#2/Protocol:Protocol/RID=N-H9AC) on GUDMAP (www.gudmap.org). Digoxygenin (DIG) labeled probes were generated following the ‘Digoxigenin-labelled Riboprobe Synthesis from a PCR-generated DNA templates’ protocol found on GUDMAP (Little Group, GUDMAP Consortium; https://www.gudmap.org/chaise/record/#2/Protocol:Protocol/RID=N-H98W). Primers used for generating probes are listed in Table S4.

### Section immunostaining

For all samples, kidneys were harvested at the necessary time point. Tissue was then fixed in 4% paraformaldehyde (PFA) in 1×PBS for 15-30 min (embryonic/postnatal) to 24 h (adult). Kidneys were washed 2× in PBS and stored for up to 2 weeks before processing. Samples were incubated in 30% sucrose in PBS overnight at 4°C. Tissue was embedded in OCT media, frozen with 100% ethanol and dry ice, and stored at −80°C for up to 2 years. Frozen tissue was sectioned at 10-12 µm. Sections were air-dried at room temperature (RT) for a minimum of 10 min. Samples were then incubated in 1× PBS for 5 min to remove OCT media. Next, sections were placed in blocking buffer (PBS with 3% bovine serum albumin, 1% donkey serum and 0.25% Triton X-100) for 20 min at RT. The blocking buffer was removed, then sections were incubated with primary antibodies in the blocking buffer for 1 h at RT. Sections were then rinsed 3× in wash buffer (PBS with 0.1% Triton X-100). Next, sections were incubated with appropriate Alexa Fluor conjugated secondary antibodies at 1:1000 (Invitrogen) in blocking buffer for 20 min. Sections were then rinsed 3× in wash buffer and mounted with Prolong Gold with DAPI (Invitrogen). Antibodies used and their dilution are listed in Table S5.

### Quantification of proliferation

Mice were injected intraperitoneally with 1.25 mg of EdU (10 mg/ml) 1.5 h before euthanasia and tissue collection. For analysis of proliferation, the following procedure was used. The tissue was fixed, then sectioned and stained as described above. The Click-It EdU kit (Thermo Fisher Scientific) was used to detect EdU^+^ cells. Images were then collected on a Zeiss 880 confocal using a 40× oil objective. The whole kidney images were created through automated tiling of the tissue section using Zen Black software. Reconstruction of the images into a single image was performed using Imaris stitcher (Bitplane). For proliferation counts, a minimum of ten nephrogenic caps were counted per section for each genotype and age. Quantitation of proliferation for nephron progenitors was determined as the number of cells colocalized for both EdU and Six2 divided by the total number of Six2^+^ cells. Ureteric bud (UB) tip niches were defined as the Ecad^+^ cells within the UB but above the ventral end of the nephrogenic cap. Quantitation for UB proliferation was determined as the number of cells within the UB niche colocalized with Ecad and EdU divided by the total number of Ecad^+^ cells within the UB niche.

### Wholemount immunostaining

The wholemount immunostaining protocol was adapted from Jafree et al. (2019) and Renier et al. (2014). All following steps were performed with rocking unless otherwise noted. Samples are collected and fixed in 4% PFA for 15-30 min (embryonic/postnatal). Tissue was washed with rocking 2× for 20 min at RT. Samples were then dehydrated in an increasing methanol/H_2_O series of 25%, 50%, 75% and 100% for 30 min or 1 h, depending on tissue size. Next, tissue was bleached overnight in 5% H_2_O_2_ (from 30% stock) in 100% methanol. Samples were then rehydrated following a reverse methanol/H_2_O series (100%, 75%, 50%, 25%, PBS) for 30 min or 1 h, depending on tissue size. Tissue was then permeabilized overnight at 4°C in PBS with 0.2% Triton X-100, 2.3% glycine and 20% DMSO. The permeabilization solution was removed, and blocking buffer (PBS with 0.2% Triton X-100, 6% donkey serum and 10% DMSO) was added; the tissue was then incubated for 1 day at RT. The blocking buffer was then removed, and primary antibody in staining solution [PBS with 0.2% Tween-20, 0.1% heparin (from 10 mg/ml stock), 3% donkey serum and 5% DMSO] was added to samples, which were then incubated for 3 days at 4°C, followed by washing 4-6× for 1 h at RT in wash buffer [PBS with 0.2% Tween-20 and 0.1% heparin (from 10 mg/ml stock)]. The appropriate Alexa fluor labeled secondary antibody in staining solution was then added (1:250) and incubated overnight at 4°C. Samples were washed 4-6× for 1 h at RT in wash buffer. Samples were then immediately moved to clearing or stored at 4°C in 1× PBS, protected from light until ready to proceed to clearing. Antibodies used and their dilution are listed in Table S5.

### Evans Blue

The full protocol for Evans Blue dye is from Honeycutt and O'Brien (2021) and is described here briefly. Animals were anesthetized to a surgical plane under isoflurane. Next, the chest cavity and peritoneum were cut to expose the diaphragm. Then 2% Evans Blue dye in 0.9% NaCl/H_2_O at 3 µl/g of body weight was injected into the left ventricle with either a 25 g needle or 31 g insulin syringe. The dye was allowed to circulate until the exposed areas (paws, tail, chin) turned blue (∼2-5 min). The animal was subsequently euthanized, and kidney was collected, rinsed briefly in PBS several times to remove excess dye, then fixed in 4% PFA for 24 h at 4°C. Samples were washed in PBS and stored at 4°C until clearing.

### Tissue clearing and imaging

This protocol was adapted from the iDISCO method of tissue clearing (Renier et al., 2014). First, embryonic and small samples were placed into agarose blocks [1% agarose in tris-acetate-EDTA (TAE)]. Adult kidneys were not embedded. All following steps were performed with rocking unless otherwise noted. These blocks or adult kidneys were dehydrated in an increasing methanol/H_2_O series of 25%, 50%, 75% and 100% for 30 min or 1 h at RT, depending on tissue size. Samples were then incubated in 66% dichloromethane (DCM)/33% methanol for 3 h at RT. Next, two rounds of incubation with 100% DCM at RT were performed. DCM was removed, and 100% dibenzyl ether (DBE) was added. Samples were incubated, with no rocking, until clear (1-24 h) and stored indefinitely in 100% DBE at either RT or 4°C, protected from light, until ready to proceed to imaging. Light-sheet imaging of whole kidneys was performed using a LaVision UltraMicroscope II (LaVision BioTec) and 3D reconstruction of images and vascular network quantification was conducted using Imaris imaging software (Bitplane).

### Quantification of wholemount 3D vasculature

To conduct quantification of renal vasculature within Imaris, a surface of the 3D image was first created to digitally render the vasculature. Next, a binary mask of the rendered surface was created using the ‘Mask channel’ function with ‘Constant inside/outside’ settings for voxel intensity set to 0 outside and 100 inside. This was necessary to render the vasculature effectively solid, so that the filament tracer module within Imaris could be implemented to develop wireframes for statistical analysis. Kidneys were batched with similar parameters per age group. Then supervised automatic tracing was performed to ensure that automatically generated paths were not duplicated, or artifacts inaccurately rendered as vasculature. As both kidneys from the same animal have highly similar statistical values, with little deviation when averaged, for most studies only one kidney per animal was needed for quantification. This method was used to quantify renal arterial trees for Evans Blue in adults and SMA and CD31 staining in the embryonic and neonatal kidneys. Cortical arterioles and vascular beds were excluded from analysis.

For graphical display of vascular patterning and imaging that were not quantified, a surface was created for the desired channel. This surface was then masked using the same procedure, but ‘Constant inside/outside’ settings for voxel intensity were set to 0 outside with the inside setting left unchecked.

### RNA-seq

Whole kidneys were collected at E15.5 from three separate *Ntn1^fl/fl^ x Foxd1^GC/+^;Ntn1^fl/+^* crosses. Three kidneys were collected of each genotype: *Foxd1^GC/+^;Ntn1^fl/+^* (heterozygous), *Foxd1^GC/+^;Ntn1^fl/fl^* (homozygous) and *Ntn1^fl/fl^* (wildtype). Kidneys were placed in RNAlater (Thermo Fisher Scientific) until all samples were collected. RNA was extracted with TRIzol (Invitrogen). RNA was sent to Cancer Genetics Inc (Morrisville, NC, USA) for sequencing and data processing. RNA samples were converted to sequencing libraries using the TruSeq Stranded mRNA kit (Illumina, 20020595). Resulting libraries were assessed for quality via analysis on a 2100 Bioanalyzer (Agilent Technologies) and quantitated by qPCR (KAPA Library Quantification; Roche, KK4824, 07960140001). Normalized and pooled libraries were sequenced on the Illumina Nextseq 550 system. Alignment and analysis were performed following a modified Tuxedo workflow. Briefly, alignment was carried out with STAR aligner followed by cufflinks/cuffmerge for assembled transcripts. FPKM was calculated with CuffDiff and analyzed with the R package. All sequencing data has been deposited in the Gene Expression Omnibus (GEO) data repository under accession number GSE245506. Heterozygous samples were used as a control, compared with the homozygous samples to account for any effects of the hemizygous *Foxd1* allele due to the knock-in of GFP-Cre into the *Foxd1* locus. A count of 5 fragments per kilobase million (FPKM) was considered minimum criteria for expression in the whole kidney RNA-seq, genes with less than this FPKM (unless downregulated compared with the reference) were excluded from gene expression analyses. Genes with ≥1.2-fold up- or downregulated and *P*≤0.05 were pasted into the Database for Annotation, Visualization and Integrated Discovery (DAVID) to obtain Gene Ontology information (Huang et al., 2009a,b).

To identify the cell populations in which the top ten up- and downregulated genes were expressed, in addition to the genes tested in qPCR, these 32 genes were analyzed for their relative enrichment within the scRNA-seq data from E18.5 kidneys (Combes et al., 2019). Heatmaps were generated in R (https://www.R-project.org/) using the ggplot2 package (Wickham, 2009). Log fold change values were obtained from Table S1, ‘lookup’ tab, of Combes et al. (2019).

### qPCR

RNA was extracted from tissue using 500 µl TRIzol reagent (Thermo Fisher Scientific, 15596026), following the manufacturer's protocol with bead homogenization using 1.4 mm zirconium-silicate spheres in a 2 ml tube (MP Bio, Lysing Matrix D, 2 ml tube, 116913050-CF). RNA concentration was then assessed using a NanoDrop Lite Spectrophotometer (Thermo Fisher Scientific, ND-LITE-PR). cDNA was generated from 1 µg of RNA using SuperScript IV Reverse Transcriptase (Thermo Fisher Scientific, 18090010), 50 µM Oligo d(T)_20_ primer and following the manufacturer's protocol. qPCR was performed on a QuantStudio 7 Flex Real-Time PCR System (Thermo Fisher Scientific, 4485688) using PowerUp SYBR Green Master Mix (Thermo Fisher Scientific, A25742) following the manufacturer's protocol. Primers used are listed in Table S4.

## Supplementary Material

10.1242/develop.201886_sup1 Supplementary information

Table S1. RNA-seq data for HET (Foxd1^GC/+^;Ntn1^fl/+^) and MUT (Foxd1^GC/+^;Ntn1^fl/fl^) kidneys

Table S2. Genes upregulated ≥ 1.2-fold and the associated Gene Ontology (GO) Biological Processes

Table S3. Genes downregulated ≥ 1.2-fold and the associated Gene Ontology (GO) Biological Processes
