# Supplementary material for: Netrin 1 directs vascular patterning and maturity in the developing kidney
Source: Development. 2023 Nov 24;150(22):dev201886. doi: 10.1242/dev.201886 (PMC10690109; doi:10.1242/dev.201886)
Supplement: Supplementary information [file develop-150-201886-s1.pdf]

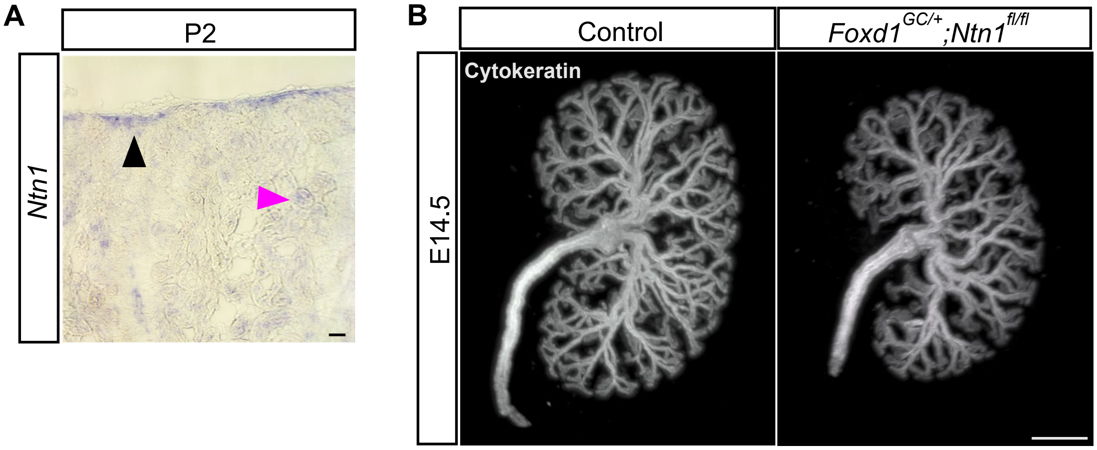

Fig. S1. *Ntn1* is expressed by the postnatal kidney and deletion of *Ntn1* from Foxd1+ progenitors results in smaller ureteric trees

A) *In situ* hybridization of sectioned postnatal day (P)2 kidneys shows *Ntn1* mRNA expression by stromal progenitors (black arrowhead) and to a lesser extent by epithelial structures (magenta arrowhead). Scale bar = 50µm. B) Wholemount immunostaining of the ureteric tree (cytokeratin, grey) at E14.5 shows that *Foxd1*$^{GC/+}$*; Ntn1*$^{fl/lf}$ kidneys are smaller than controls at this stage. Scale bar = 200µm.

Development • Supplementary information

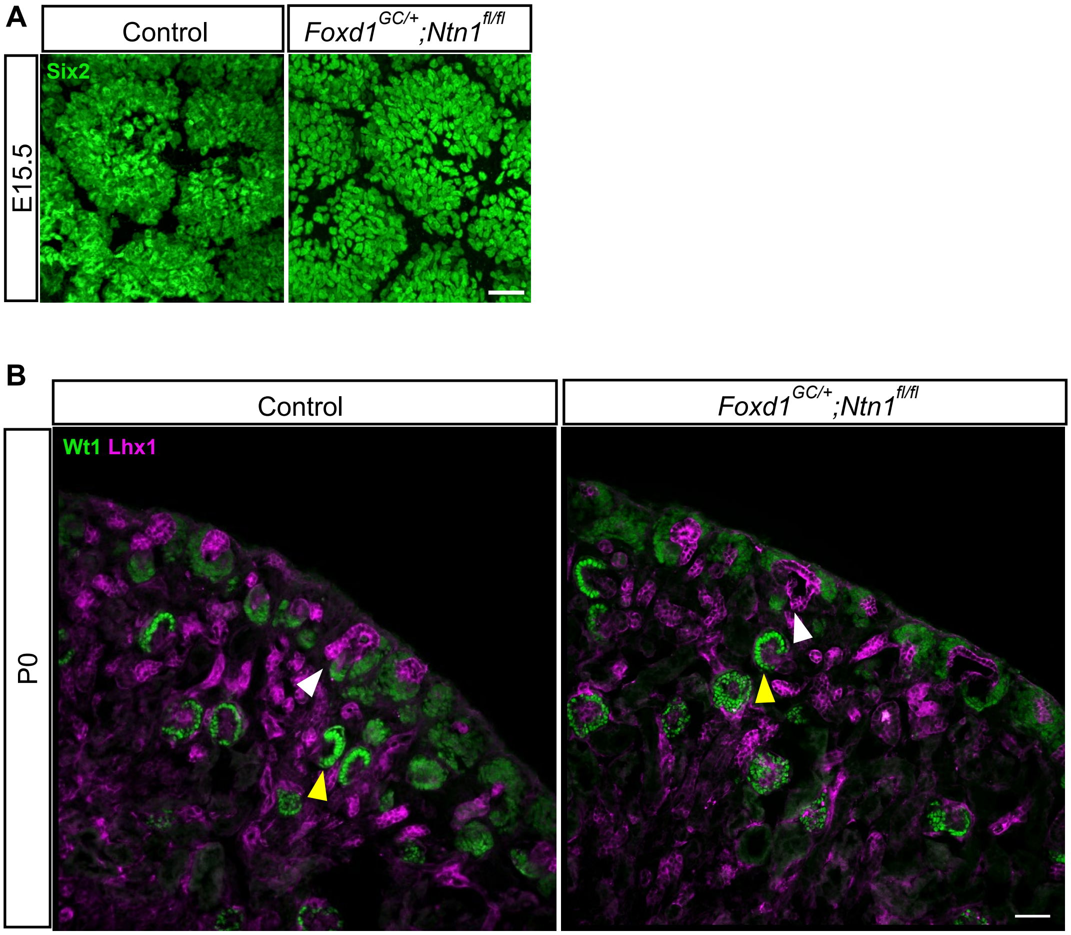

Fig. S2. Nephron progenitor niches appear normal and maintain differentiation capacity in *Foxd1$^{GC/+}$; Ntn1$^{fl/fl}$* kidneys

A) Wholemount immunostained kidney surface at E15.5 shows normal organization and nephron progenitors (Six2+, green) in *Foxd1$^{GC/+}$; Ntn1$^{fl/fl}$* mice. Scale bar = 20µm. B) Sections of kidneys from *Foxd1$^{GC/+}$; Ntn1$^{fl/fl}$* mice and controls show the normal differentiation of nephrons throughout development as labeled by Wt1 (green, yellow arrowhead, nephron progenitors and developing podocytes) and Lhx1 (magenta, white arrowhead, ureteric collecting duct system and developing nephrons). Scale bar = 50µm.

Development • Supplementary information

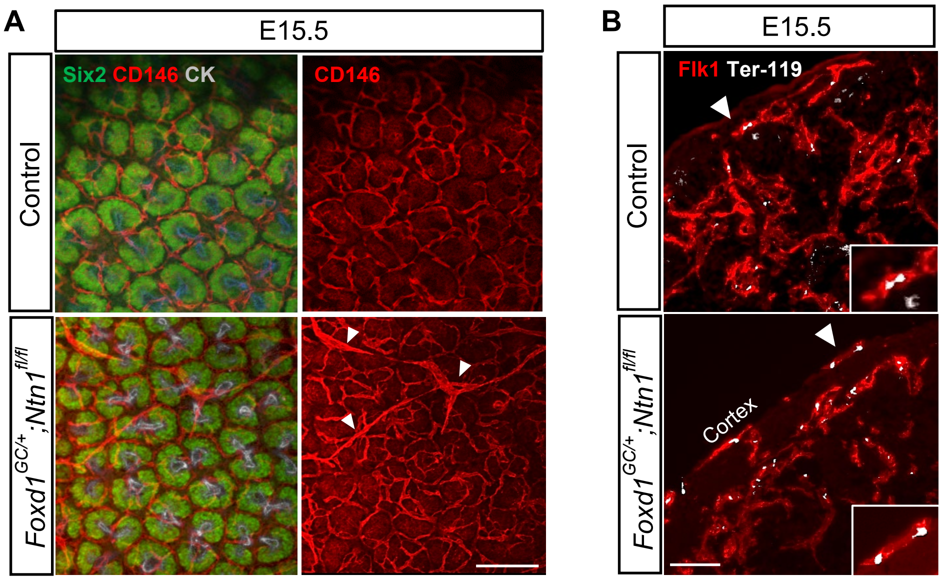

Fig. S3. *Foxd1$^{GC/+}$; Ntn1$^{fl/fl}$* kidneys display vascular patterning defects around nephron progenitor niches and vessels contain erythroid cells

A) Confocal images of the wholemount immunostained kidney surface at E15.5 showing ectopic vessels (CD146+, red) next to nephron progenitor (Six2+, green) and ureteric tip (cytokeratin, CK+, grey) niches in *Foxd1$^{GC/+}$; Ntn1$^{fl/fl}$* kidneys. Scale bar = 100μm. B) Sections of E15.5 kidneys show Ter-119+ erythroid cells (white) are present in the vasculature (Flk1, red) of both control and *Foxd1$^{GC/+}$; Ntn1$^{fl/fl}$* kidneys. Insets show higher magnification views. Scale bar = 50μm.

Development • Supplementary information

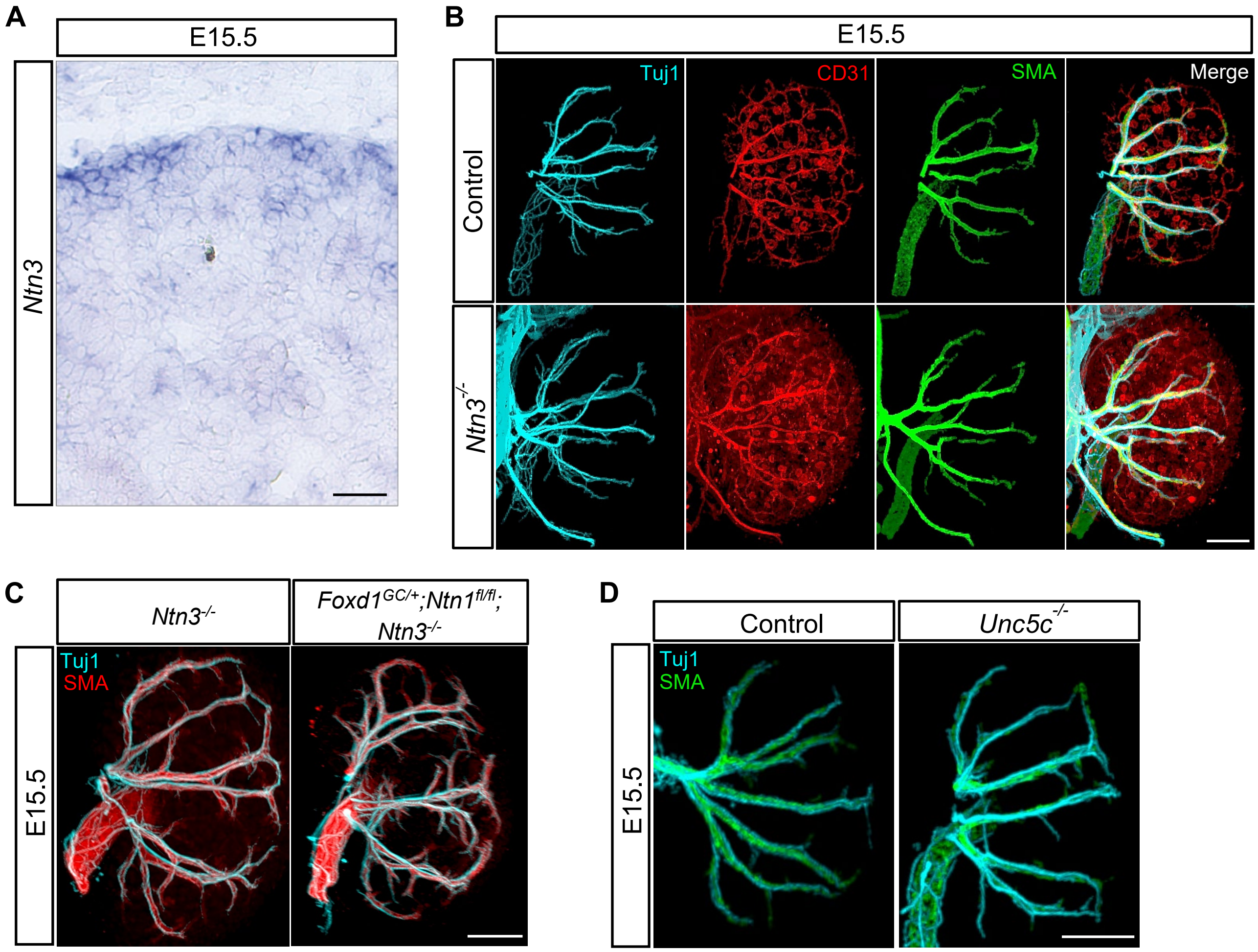

Fig. S4. Deletion of *Ntn3* or *Unc5c* does not cause significant vascular patterning defects nor exacerbate the *Foxd1$^{GC/+}$;Ntn1$^{fl/fl}$* phenotype with co-deletion of *Ntn3*

A) *In situ* of *Ntn3* at E15.5 on wildtype sections. A similar pattern to *Ntn1* is observed. Scale bar = 50μm. B) Wholemount immunofluorescent images of *Ntn3* knockout kidneys showing no significant differences in vascular (SMA, green; CD31, red) or neuronal (Tuj1, cyan) patterning. Scale bar = 300μm. C) Wholemount immunofluorescent images show that double knockout *Foxd1$^{GC/+}$; Ntn1$^{fl/fl}$;Ntn3$^{-/-}$* kidneys do not show any exacerbated vascular patterning phenotypes (SMA, red; Tuj1, cyan). Scale bar = 400μm. D) Wholemount immunofluorescent images show that vascular patterning is largely normal in *Unc5c* knockouts (Tuj1, cyan; SMA, green). Scale bar = 300μm.

Development • Supplementary information

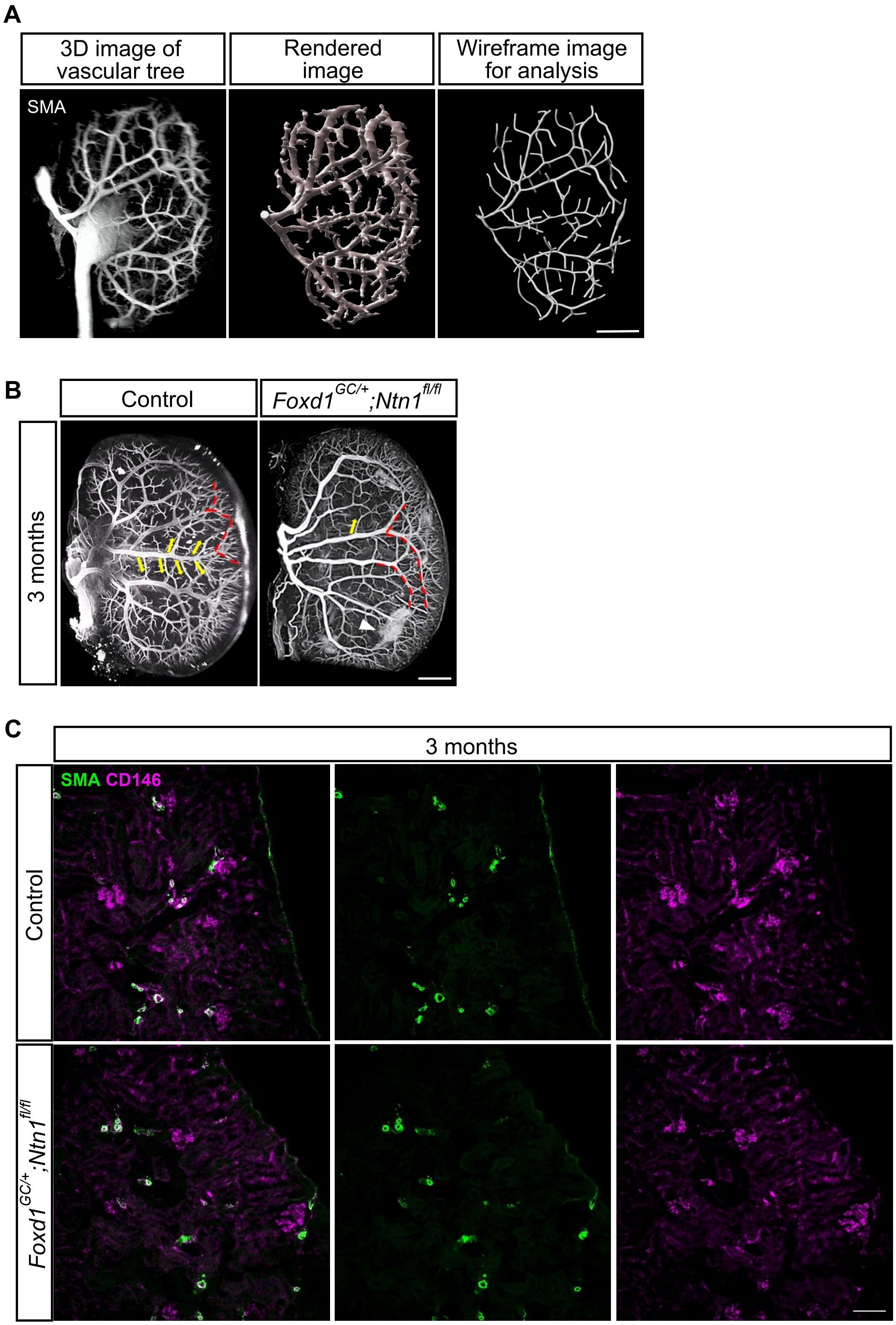

Fig. S5. Depictions of rendering used for vascular (arterial tree) analysis and how the patterning defects persist at 3 months

Development • Supplementary information

A) Micrographs showing the original image of vascular trees, the Imaris rendered image, and resulting wireframe image used for analysis. Scale bar = 500μm B) Images of 3-month-old adult kidneys perfused with Evans blue showing the persistence of vascular patterning defects such as meandering vessels (red dotted lines) and reduced branching (yellow arrows). Clouds of Evans blue are occasionally observed (white arrowhead), potentially representing vascular leakage. Scale bar = 1000μm. C) Images of sectioned 3-month-old kidneys showing the endothelium (CD146, magenta) and smooth muscle coated vasculature (SMA, green) at the cortex. In section, the vasculature appears qualitatively equivalent between control and *Foxd1*$^{GC/+}$*; Ntn1*$^{fl/fl}$ animals. Scale bar = 50μm.

Development • Supplementary information

Table S1. RNA-seq data for HET (*Foxd1*$^{GC/+}$;*Ntn1*$^{fl/+}$) and MUT (*Foxd1*$^{GC/+}$;*Ntn1*$^{fl/fl}$) kidneys

Available for download at
https://journals.biologists.com/dev/article-lookup/doi/10.1242/dev.201886#supplementary-data

Table S2. Genes upregulated $\geq$ 1.2-fold and the associated Gene Ontology (GO) Biological Processes

Available for download at
https://journals.biologists.com/dev/article-lookup/doi/10.1242/dev.201886#supplementary-data

Table S3. Genes downregulated $\geq$ 1.2-fold and the associated Gene Ontology (GO) Biological Processes

Available for download at
https://journals.biologists.com/dev/article-lookup/doi/10.1242/dev.201886#supplementary-data

Table S4. List of primers used for genotyping, *in situ* hybridization probes, and qPCR

**Genotyping Primers**	
Ntn1_F	GGCAGTGAATGTGTTTCCGTT
Ntn1_R	ATCGCGGGATAGTGGGTTTC
Foxd1_WT_F	CTCCTCCGTGTCCTCGTC
Foxd1_Mut_F	GGGAGGATTGGGAAGACAAT
Foxd1_Common_R	TCTGGTCCAAGAATCCGAAG
Ntn3_Internal(WT)_F	GGCCTGTGGTCTGGTTACAG
Ntn3_Internal(WT)_R	TAGCCTGGGGACTTCTGACC
Ntn3_External(deletion)_F	CACCTCCCAGCAGCAAGTAAC
Ntn3_External(deletion)_R	ACGCAGAGTAGCGGACTAGG
TrkA_1	TGTACGGCCATAGATAAGCAT
TrkA_2	TGCATAACTGTGTATTTCAC
TrkA_3	CGCCTTCTTGACGAGTTCTTCTG
Unc5c_WT_F	CACTCTATGGAAATGGCTGAAT
Unc5c_WT_R	GTCCTCCAATCCAAGAACTG
Unc5c_Mut_F	CAGGAGAAGATACATTTAACCAC
Unc5c_Mut_R	GACAGAAGAGCATAGCATTCAC
***In situ* primers**	
Ntn1 F	CTTCCTCACCGACCTCAATAAC
Ntn1 (SP6) R	GCGATTTAGGTGACACTATAGTTGTGCCTACAGTCACACACC
Ntn3 F	GGCACGCTCTCCGCTGCA
Ntn3 (T7) R	TAATACGACTCACTATAGGGAGTGGCTGAGTAGGAGTA
Unc5c F	ATGAGGAAAGGTCTGAGGGCGACAG
Unc5c (T7) R	TAATACGACTCACTATAGGGAGAGAGTTGAAGGTACCAA
Unc5b F	TCAAGTGTAATGGCGAGTG
Unc5b (T7) R	TAATACGACTCACTATAGGGGCCTCCATTCACATAGACGA
**qPCR primers**	
Unc5b_F	CGGGACGCTACTTGACTCC
Unc5b_R	GGTGGCTTTTAGGGTCGTTTAG
Parvb_F	AAGGACGAGTCTTTCTTGGGC
Parvb_R	GGGGCCATTGGAGAGTTGAT
Notch1_F	GATGGCCTCAATGGGTACAAG
Notch1_R	TCGTTGTTGTTGATGTCACAGT
Col15a1_F	CTCGCGGGTTACATAAGGCT

Development • Supplementary information

Col15a1_R	GTAGAGGATAACCCGCTGGC
Ltbp4_F	CTGGGTGTCGCTATTGGTG
Ltbp4_R	GTTGTGACAGATCAAGGGACAT
Actg2_F	CCGCCCTAGACATCAGGGT
Actg2_R	TCTTCTGGTGCTACTCGAAGC
Crabp1_F	CAGCAGCGAGAATTTCGACGA,
Crabp1_R	CGCACAGTAGTGGATGTCTTGA
Vim_F	CGTCCACACGCACCTACAG
Vim_R	GGGGGATGAGGAATAGAGGCT
Col4a1_F	CTGGCACAAAAGGGACGAG
Col4a1_R	ACGTGGCCGAGAATTTCACC
Tgfb1_F	CTCCCGTGGCTTCTAGTGC
Tgfb1_R	GCCTTAGTTTGGACAGGATCTG
Hey1_F	GCGCGGACGAGAATGGAAA
Hey1_R	TCAGGTGATCCACAGTCATCTG
Acta2_F	GTCCCAGACATCAGGGAGTAA
Acta2_R	TCGGATACTTCAGCGTCAGGA

Table S5. List of antibodies utilized for section and wholemount immunostaining

Antibody	Host /Isotype	Concentration (section)	Concentration (wholemount)	Company	Catalog #
Six2	Rabbit	1:1000	1:250	ProteinTech	11562-1-AP
Netrin-1	Chicken	1:100	NA	Abcam	ab39370
CD31	Rat	1:100	1:50	BD Biosciences	550274
SMA-488	Mouse IgG2a	NA	1:250	Invitrogen	53-9760-82
Cytokeratin	Mouse IgG1	1:500	1:250	Sigma	C2931
Ecad	Rat	1:500	1:250	Fisher	131900
AlexaFluor Azide Dye (EdU)	NA	1:500	NA	ThermoFisher	A10266
CD146 (Mcam)	Rat	1:1000	1:250	BioLegend	164701
Tubb3 (Tuj1)	Mouse IgG2a	1:1000	1:250	BioLegend	801202
Tubb3-594 (Tuj1)	Mouse IgG2a	1:1000	1:250	BioLegend	818001
Flk1	Goat	1:250	NA	R&D Systems	AF644
TER119	Rat	1:250	NA	BD Biosciences	553670
Lhx1	Mouse IgG1	1:100	NA	DSHB	4F2
Wt1	Rabbit	1:500	NA	Abcam	ab89901

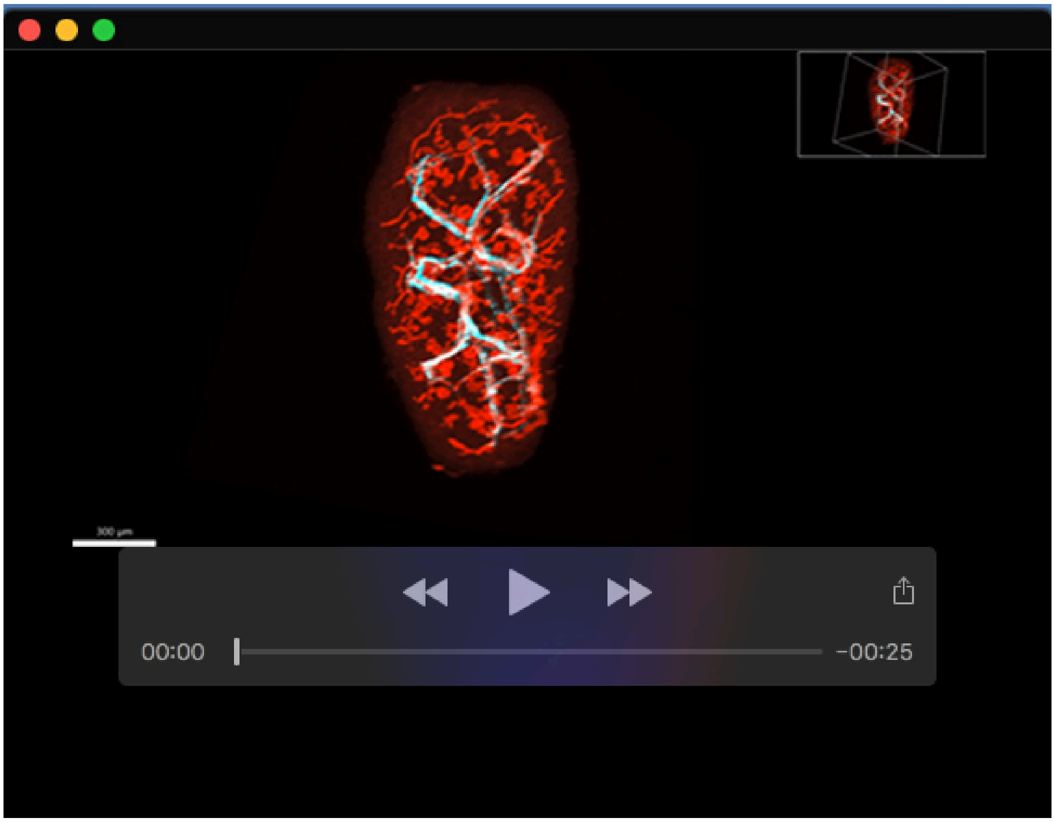

Movie 1. Video associated with Fig. 4C showing vascular patterning of a control kidney. Tuj1+ nerves: cyan; CD31+ endothelium: red

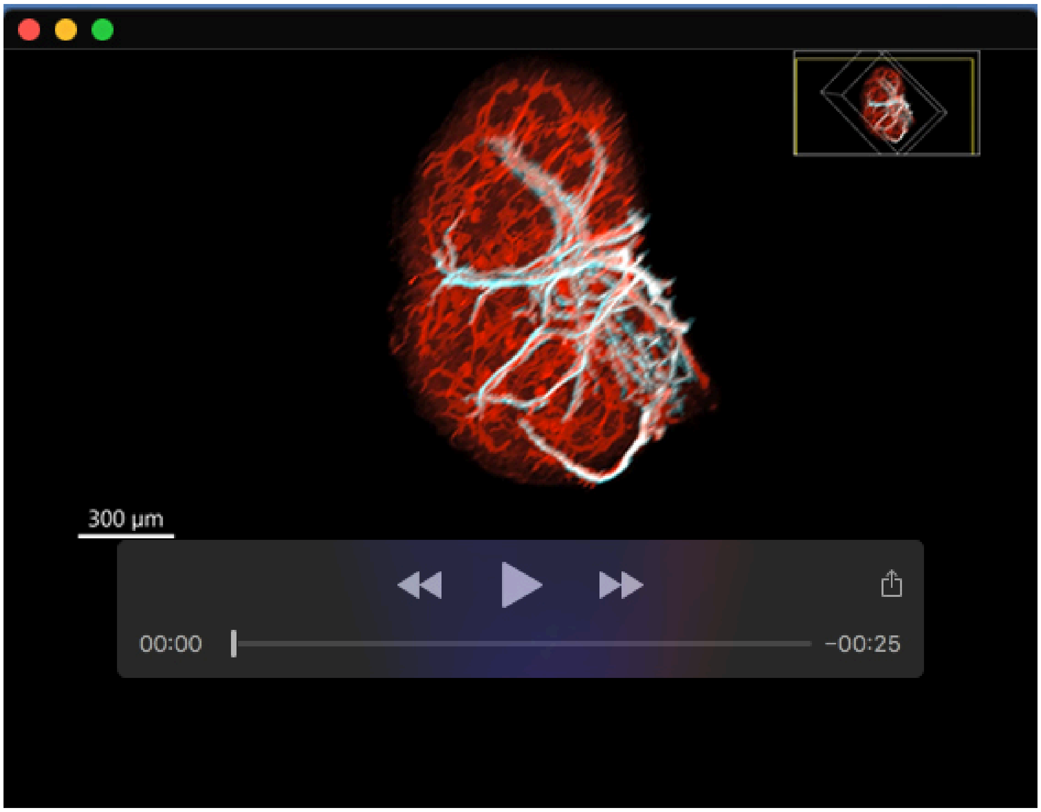

Movie 2. Video associated with Fig. 4C showing vascular patterning of a *Foxd1*$^{GC/+}$;*Ntn1*$^{fl/fl}$ kidney. Tuj1+ nerves: cyan; CD31+ endothelium: red

Development • Supplementary information

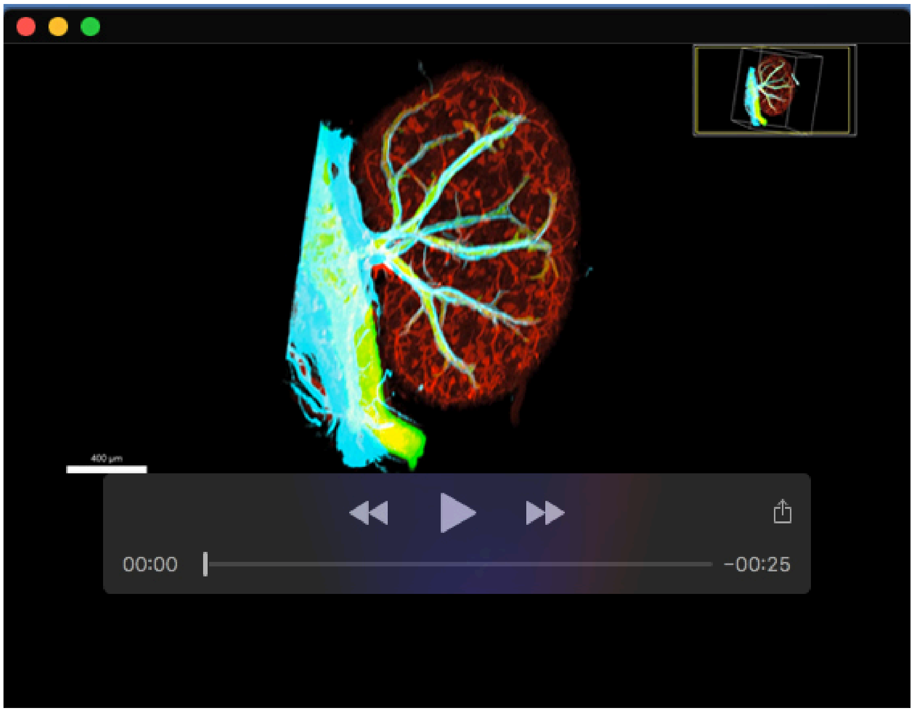

Movie 3. Video associated with Fig. 5A showing vascular patterning of a control kidney. Tuj1+ nerves: cyan; CD31+ endothelium: red, SMA+ mural cells: green

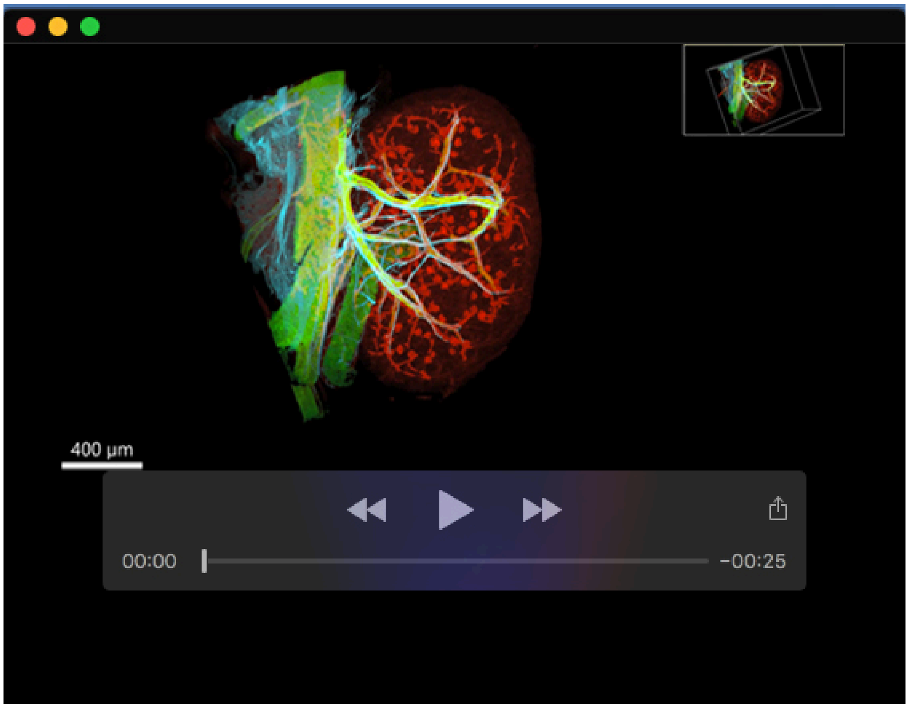

Movie 4. Video associated with Fig. 5A showing vascular patterning of a *Foxd1*$^{GC/+}$;*Ntn1*$^{fl/fl}$ kidney. Tuj1+ nerves: cyan; CD31+ endothelium: red, SMA+ mural cells: green

Development • Supplementary information
