# Supplementary material for: Netrin 1 directs vascular patterning and maturity in the developing kidney
Source: Development. 2023 Nov 24;150(22):dev201886. doi: 10.1242/dev.201886 (PMC10690109; doi:10.1242/dev.201886)
Supplement: Supplementary file 1 [file develop-150-201886_review_history.pdf]

Netrin 1 directs vascular patterning and maturity in the developing kidney

Samuel E. Honeycutt, Pierre-Emmanuel Y. N'Guetta, Deanna M. Hardesty, Yubin Xiong, Shamus L. Cooper, Matthew J. Stevenson and Lori L. O'Brien
DOI: 10.1242/dev.201886

Editor: Liz Robertson

Review timeline

Original submission:	18 April 2023
Editorial decision:	1 June 2023
First revision received:	7 September 2023
Accepted:	2 October 2023

Original submission

First decision letter

MS ID#: DEVELOP/2023/201886

MS TITLE: Netrin-1 directs vascular patterning and maturity in the developing kidney

AUTHORS: Samuel E Honeycutt, Pierre-Emmanuel Y N'Guetta, Deanna M Hardesty, Yubin Xiong, Shamus L Cooper, and Lori L O'Brien

I have now received all the referees' reports on the above manuscript, and have reached a decision. The referees' comments are appended below, or you can access them online: please go to BenchPress and click on the 'Manuscripts with Decisions' queue in the Author Area.

As you will see, the referees express considerable interest in your work, but have some significant criticisms and recommend a substantial revision of your manuscript before we can consider publication. If you are able to revise the manuscript along the lines suggested, which may involve further experiments, I will be happy receive a revised version of the manuscript. Your revised paper will be re-reviewed by one or more of the original referees, and acceptance of your manuscript will depend on your addressing satisfactorily the reviewers' major concerns. Please also note that Development will normally permit only one round of major revision. If it would be helpful, you are welcome to contact us to discuss your revision in greater detail. Please send us a point-by-point response indicating your plans for addressing the referee's comments, and we will look over this and provide further guidance.

As your paper was submitted alongside the one from the Cleaver lab, the Reviewers have indicated that they think it would be appropriate if your laboratories could discuss and share the data contained in your respective papers and address any discrepancies in interpretation. Ondine's paper has also been returned for revision to address the concerns raised by the Reviewers. You might also want to consult with each other to ensure that the revised versions of your papers are re-submitted at the same time.

Please attend to all of the reviewers' comments and ensure that you clearly highlight all changes made in the revised manuscript. Please avoid using 'Tracked changes' in Word files as these are lost in PDF conversion. I should be grateful if you would also provide a point-by-point response detailing how you have dealt with the points raised by the reviewers in the 'Response to Reviewers' box. If you do not agree with any of their criticisms or suggestions please explain clearly why this is so.

© 2023. Published by The Company of Biologists under the terms of the Creative Commons Attribution License (https://creativecommons.org/licenses/by/4.0/).

Reviewer 1

Advance summary and potential significance to field

The article by Honeycutt et al. details how Netrin-1 plays an important role in vascular patterning and maturation in the developing kidney. The authors use a combination of genetic deletion and advanced imaging couples with molecular analysis to determine an important developmental role of Netrin-1 in the renal stromal cells. The concept of the vessels and the nerves interacting and that in this context it is the vessels that are leading the nerves is novel and significant. The spatial imaging of the vessels and the nerves is also a strength and interesting to the field.

Comments for the author

The authors have done an excellent job of describing the phenotype that arises from the Netrin-1 knockout animals. However there is limited mechanistic insight and some of the analysis are quite superficial. Further investigation is required before this reaches a critical threshold.

Major criticisms:
1. In figure 1 the overall size of the kidneys is altered at E15.5 in the mutants compared to the controls.
However, it is not clear if this is just an alteration in overall embryo size. Measurement of embryo length/weight would be important. Further, it is known that the lungs of animals also have high expression of Foxd1 was there any change in lung size?
2. It is not clear whether the nephron progenitor persistence out to P4 is due to a delay and underdevelopment rather than a specific nephron progenitor defect. Do these nephron progenitors persist at later postnatal time points. Could the nephron progenitors be isolated and their differentiation potential characterized in vitro compared to control progenitors?
3. It is interesting that the vascular patterning largely resolves in the mutants when adult animals are studied.
This suggests that early vascular patterning and organization is largely dispensable in particular to Netrin-1 deficiency. Further comment and discussion of this is warranted.
4. The RNA sequencing data seems very premature and preliminary. A better analysis should at the very least included control tissues. Further to this validation of the gene lists using PCR and or in situ is required.

Minor criticisms:
1. Some of the figure numbers that appear throughout the manuscript do not match with the description including "Fig 3c" on page 8 and "Fig 5d" on page 12.

Reviewer 2

Advance summary and potential significance to field

Netrin-1, secreted by the renal stromal population plays a role in controlling smooth muscle investment of the renal arterial bed.

Comments for the author

An understanding of the molecular mechanisms controlling organ specific vascular patterning is essential for the generation of functional, stem cell derived tissues.
However, this aspect of vascularization during embryogenesis lags far behind the mechanisms controlling vessel formation. Honneycutt et al. tackle this important, yet poorly understood process in the developing kidney.

The study is focused on the role of the guidance molecule,netrin-1, in murine kidney development. Netrin-1 is localized at both the mRNA and protein levels and ablated from the Foxd-1 stromal population by well-established genetic techniques. The resulting conditional netrin-1 null mice

© 2023. Published by The Company of Biologists under the terms of the Creative Commons Attribution License (https://creativecommons.org/licenses/by/4.0/).

exhibit a complex renal phenotype but Honeycutt et. al do an excellent job in documenting it by quantitative and functional criteria. The study conclusively demonstrates that netrin-1 plays a direct role in controlling the temporal and spatial association of vascular mural cells to the renal vascular bed. Although the cause of the small kidney size cannot be attributed to a direct role for nephrin-1 in renal epithelial morphogenesis, the discussion of the manuscript well addresses different possibilities.

Reviewer 3

Advance summary and potential significance to field

Honeycutt et al. investigate the role of the secreted guidance cue Netrin1 in the developing kidney. They used a stromal cell Cre line to generate mice harboring a stromal cell specific Ntn1 knockout, and showed that mutant mice develop hypoplastic kidneys at postnatal stages, but can live to adulthood and are described as healthy and devoid of renal defects. The developmental delay of the mutant kidneys is attributed to transient kidney nephrogenesis and arteriogenesis defects. The phenotypes presented overall match the ones shown in the accompanying manuscript by Luo et al, and support that stromal cell derived Netrin1 developmentally regulates morphogenesis of nephrons and renal arterial patterning and maturation. Mechanistically, the authors investigate one candidate Netrin1 receptor, Unc5c, which is expressed in nephron progenitors, but fail to observe defects in kidney development of these mutants. They also address potential redundancy of Netrin1 with Netrin3, but fail to observe any phenotypes in Ntn3 deficient mice. Finally, because they observe defective patterning of arteries and adjacent nerves, they address whether deleting TrkA dependent nerve growth affects vessel patterning in Ntn1 mutants, but find that ectopic arteries persist in Ntn1 mutants in the absence of axons, suggesting effects on the endothelium that remain however uninvestigated. Bulk RNA sequencing of wildtype and Ntn1 mutant kidneys is reported at E15, the genes regulated by Ntn1 deficiency are different from the ones reported in Luo et al, notably KLF4 is not downregulated in this dataset. No validation of this dataset is provided. Overall, the data support important and complex developmental roles of Netrin1 in kidney morphogenesis, and provide a starting point for understanding the mechanism of action, which remains unclear.

Comments for the author

1- The delay in cessation of nephrogenesis seen in in Foxd1;Ntn1 kidneys is interesting but remains elusive. The authors claim that the delay in cessation of nephrogenesis seen in Foxd1;Ntn1 mice is independent of the Netrin1 receptor Unc5C. To make this claim, they should investigate Six2+ nephron progenitor cells in Unc5B KO kidneys at P4 and not at P0 where this population is still present in control kidneys. Also please show kidney size of Unc5c mutants at pre- and postnatal stages, and provide a table with the number of kidneys analyzed per genotype and per stage.

2. Please explain rationale behind the TRKA deletion experiment in Fig.3. The text and its interpretation are hard to follow here.

2- While non-significant in their quantification, the 2month old Foxd1;Ntn1 KO kidney seems to have strongly decreased vascular density, and the image provided does not match their quantification. Furthermore, Evans blue only highlights perfused vessel, can the authors co-stain with SMA and CD31 to analyze differences in vascular development.

3- To validate bulk RNA sequencing data at least to some extent, please show if ectopic SMA positive cells are seen in the cortex of the mutant kidneys. It remains unclear if this phenotype is shared with Luo et al or not.

First revision

Author response to reviewers' comments

© 2023. Published by The Company of Biologists under the terms of the Creative Commons Attribution License (https://creativecommons.org/licenses/by/4.0/).

We would like to thank the reviewers for taking the time to review our original manuscript critically and thoroughly. We have taken into account all the reviewer comments and revised our manuscript accordingly to address each reviewer's concerns to the best of our ability. These changes have improved our manuscript, helping to solidify several of our findings and to gain greater insights into how netrin-1 signaling mediates vascular patterning and maturity in the developing kidney and overall kidney development. We hope the reviewers will consider our findings complementary to those of Dr. Ondine Cleaver and her group, and further consider our manuscripts for co-publication in *Development*.

* For all significant changes to the manuscript text file, the new/changed text is blue to help direct the reviewer's attention to these changes.

Reviewer-specific responses

Reviewer 1

Reviewer 1 Advance Summary and Potential Significance to Field

The article by Honeycutt et al. details how Netrin-1 plays an important role in vascular patterning and maturation in the developing kidney. The authors use a combination of genetic deletion and advanced imaging couples with molecular analysis to determine an important developmental role of Netrin-1 in the renal stromal cells. The concept of the vessels and the nerves interacting and that in this context it is the vessels that are leading the nerves is novel and significant. The spatial imaging of the vessels and the nerves is also a strength and interesting to the field.

Reviewer 1 Comments for the Author

The authors have done an excellent job of describing the phenotype that arises from the Netrin-1 knockout animals. However there is limited mechanistic insight and some of the analysis are quite superficial. Further investigation is required before this reaches a critical threshold.

We thank this reviewer for their positive comments on our findings and our characterization of the *Nnt1* conditional knockout phenotype. We appreciate the constructive criticisms and have addressed the concerns to the best of our ability as outlined below. While we still lack a well-defined mechanism due to the complexity of the phenotypes, we hope that our additional analyses provide deeper insights into how netrin-1 signals mediate vascular patterning, maturity, and kidney development.

Major criticisms:

1. In figure 1 the overall size of the kidneys is altered at E15.5 in the mutants compared to the controls. However, it is not clear if this is just an alteration in overall embryo size. Measurement of embryo length/weight would be important. Further, it is known that the lungs of animals also have high expression of Foxd1 was there any change in lung size?

Thank you for this suggestion on additionally assessing body length and weight. We have now added measurements of body weights and lengths for all three stages which we assessed- E13.5, E15.5, and P0 (Fig. 1E). We find no significant difference between body weights and lengths at any stages, while the differences in kidney length at E15.5 and P0 remain statistically significant ($p<0.001$).

In respect to the lungs, we believe the reviewer may have meant *Ntn1*, which is known to be expressed in the developing lungs (Liu et al., PMID: 15186747). We've included here an *in situ* image of *Ntn1* showing expression in the lung at E14.5 (Reviewer Fig. 1A). We assessed the expression of *Foxd1* in the lung but do not see any expression at E14.5 by *in situ* (Reviewer Fig. 1B). We also assessed the localization of Foxd1+ cells at E11.5, at the earliest stages of lung branching. However, we do not see any significant localization of Foxd1+ cells in the area of the developing lung (Reviewer Fig. 1C). Since we are using a *Foxd1* locus driven Cre to delete *Ntn1*, both would need to be expressed in the same cell during lung development. With the lack of any significant Foxd1 expression/localization in the developing lung, we do not expect it to delete *Ntn1* in the lung, and therefore do not expect any significant lung phenotypes. Although not pictured here, we have not observed any gross morphological differences in the lungs of *Foxd1*$^{GC/+}$; *Nnt1*$^{fl/fl}$ animals.

© 2023. Published by The Company of Biologists under the terms of the Creative Commons Attribution License (https://creativecommons.org/licenses/by/4.0/).

NOTE: Figure provided for reviewer has been removed. It showed *Ntn1* set EB1773 and *Foxd1* set MH2269 from Visel, A., Thaller, C. and Eichele G. (2004). GenePaint.org: an atlas of gene expression patterns in the mouse embryo. Nucleic Acids Res. 32 (Suppl. 1), D552-D556. doi:10.1093/nar/gkh029. We have removed unpublished data that had been provided for the referees in confidence.

NOTE: We have removed unpublished data that had been provided for the referees in confidence.

Reviewer Figure 1
A) *Ntn1 in situ* at E14.5 showing lung and kidney expression. B) *Foxd1 in situ* at E14.5 showing kidney expression but no lung expression. C) Wholemount immunostain of E11.5 *Foxd1*$^{GC/+}$ embryos with a GFP antibody to show the localization of Foxd1+/GFP-Cre+ cells. We do not observe any Foxd1+/GFP-Cre+ cells in the region of the developing lung.

2. It is not clear whether the nephron progenitor persistence out to P4 is due to a delay and underdevelopment rather than a specific nephron progenitor defect. Do these nephron progenitors persist at later postnatal time points. Could the nephron progenitors be isolated and their differentiation potential characterized in vitro compared to control progenitors?

We agree that the reason for the persistence of the nephron progenitors is not clear. We have now assessed P5 kidneys for the persistence of Six2+ nephron progenitors to determine if nephrogenesis is proceeding. At P5, we find that the majority of Six2+ cells have differentiated and only small clusters of Six2+ cells can be found (Six2 protein is maintained for a short period as the nephron progenitors differentiate). Images of the P5 kidneys are now included in Fig. 2D. We have also added the following to the manuscript:

Pg. 7: "Strikingly though, at P4, *Foxd1*$^{GC/+}$*; Ntn1*$^{fl/fl}$ kidneys retained small clusters of Six2+ cells whereas controls had exhausted their Six2+ population as expected (Fig. 2C). However, the Six2+ cells maintained differentiation capacity and by P5 were nearly exhausted (Fig. 2D)."

We appreciate the suggestion to isolate the progenitors to assess differentiation potential, as it would be informative to know whether this is altered in the *Ntn1* conditional knockouts. However, the isolation and culture of nephron progenitors is quite challenging and not a technique that is an established standard in our lab (we have tried in the past and have been unable to maintain cultures). Protocols for culturing nephron progenitors have been established (Brown et al., PMID: 26190145), but when isolated they need a myriad of growth factors to maintain them (prior to the ability to differentiate them *in vitro*) and this may 'reset' any progenitor phenotypes that are present *in vivo*. Since nothing has been genetically modified in the nephron progenitors in our knockouts, there are likely no permanent changes to their programs. It has been established that while there are differences between 'young' and 'old' progenitors in the nephron progenitor niche, 'old' cells can be rejuvenated by being around 'young' cells in part due to growth factors they release (Chen et al., PMID: 26460946). Since similar growth factors are utilized in culture, we predict that as above normal nephron progenitor signatures would likely be 'reset', and the differentiation capacity normalized between the genotypes in our studies. Therefore, we did not predict that this experiment would provide additional insights that would help us sort out any differences in differentiation capacity. In lieu of this experiment, we have added images of P0 kidney sections from controls and *Foxd1*$^{GC/+}$*; Ntn1*$^{fl/fl}$ animals which show the presence of differentiating nephrons in both genotypes, with no qualitative differences (Fig. S2A). We have added the following to the manuscript in reference to this addition:

Pgs. 6-7: "Analysis of nephron progenitor niches at E15.5 and P0 revealed no obvious differences in their organization, suggesting normal progression of nephrogenesis (Fig. 2B, Fig. S2A). Additionally, nephrogenesis qualitatively appeared normal in *Foxd1*$^{GC/+}$*; Ntn1*$^{fl/fl}$ P0 kidneys as assessed by Lhx1 and Wt1 immunostaining and their respective labeling of the differentiating nephron segments (Lhx1-distal, Wt1-proximal/podocyte; Fig. S2B)."

The new P5 and P0 data still do not specifically sort out the developmental delay vs. altered differentiation capacity, as even though the progenitors are capable of differentiation they may do so at a slower rate. However, little is known about how differentiation of nephrons

© 2023. Published by The Company of Biologists under the terms of the Creative Commons Attribution License (https://creativecommons.org/licenses/by/4.0/).

feeds back onto overall kidney development, which could lead to an overall delay. Similarly, we don't know much about how delays in vascular maturation may influence overall kidney development and progenitor differentiation capacity. Therefore, without more defined ways of parsing out the 'chicken and egg' of all the phenotypes, these remain open questions. We have expanded our discussion and address these aspects of the phenotypes in the section "*Potential mechanisms behind Foxd1$^{GC/+}$; Ntn1$^{fl/fl}$ hypoplasia.*"

3. It is interesting that the vascular patterning largely resolves in the mutants when adult animals are studied. This suggests that early vascular patterning and organization is largely dispensable in particular to Netrin-1 deficiency. Further comment and discussion of this is warranted.

We agree with the reviewer that it is interesting that the overall vascular pattern largely resolves in *Foxd1$^{GC/+}$; Ntn1$^{fl/fl}$* mice as the network grows and more vessels are formed (and need to pack into the tissue). However, we do still observe abnormal vessels such as those that appear longer/meandering in the *Foxd1$^{GC/+}$; Ntn1$^{fl/fl}$* adult kidneys (Fig. 5C and S5B), as well as a possible reduction in branching along some arteries (which could be compensated with additional branching at other spots or of other arteries). Our quantitative metrics do not take into account where the branching occurs or how long any individual branch is, so there may be patterning defects that are not identified in our quantitative metrics or can be easily visualized in adults where the vascular network is much more complex. Therefore, we have tempered any statements in our manuscript on the phenotypes resolving, as we cannot say this definitively-just that quantitative metrics suggest the networks are more equivalent. Additionally, we need to do more testing in the adults to validate whether the networks are physiologically equivalent under both normal conditions and injury/stress/aging and this is a focus of future studies. The Discussion has been amended as suggested:

Pg. 18: "While the *Foxd1$^{GC/+}$; Ntn1$^{fl/fl}$* adults appear to have attained equivalent vascular metrics, the metrics we assess do not provide information on where the branching occurs or the length of any individual branch. Therefore, there may be patterning defects that are not identified in our quantitative metrics or which can be easily visualized in adults where the vascular network is much more complex. We do observe some longer arterial branches in the adult kidneys as well as reduced branching along arteries (Figs. 5C, S5B). Therefore, while our basic quantitative assessment shows that branch numbers, branch points, and total length are roughly equivalent between controls and *Foxd1$^{GC/+}$; Ntn1$^{fl/fl}$* kidneys is does not inform of different patterns that may still generate similar metrics. To resolve this, we will need to develop more refined metrics that can assess differences in vascular network spatial orientations."

4. The RNA sequencing data seems very premature and preliminary. A better analysis should at the very least included control tissues. Further to this validation of the gene lists using PCR and or in situs is required.

We agree with the reviewer's assessment and have reanalyzed our bulk RNA-seq data with greater stringency. Our comparison was to heterozygous controls (*Foxd1$^{GC/+}$; Ntn1$^{fl/fl}$*) to account for any influence of the *Foxd1^{GC}* allele. This allele is a knock-in, effectively removing *Foxd1* and inserting a *GFP-Cre* into the locus. Therefore, in our *Ntn1* conditional knockouts we are also missing one allele of *Foxd1* which could have an influence on the phenotype. Therefore, our control tissue for comparison is also missing a copy of *Foxd1*. While the *Foxd1^{GC}* alone would be a better control than a heterozygous mouse, our optimized breeding strategy to obtain het and homozygous animals did not produce *Foxd1$^{GC/+}$* mice and therefore we did not perform RNA-seq on these mice. However, for our qPCR analyses as described below we use the *Ntn1$^{fl/fl}$* as a control and see similar trends in up- and downregulation for the genes analyzed, suggesting *Ntn1$^{fl/fl}$* and *Foxd1$^{GC/+}$; Ntn1$^{fl/fl}$* are relatively equivalent controls and the one missing *Foxd1* allele likely has minimal influence on the phenotypes.

Our prior gene ontology analyses utilized all genes that were $\geq$1.2 fold up- or downregulated and in our revision we have now sorted these genes by significance (p-value) and only performed gene ontology analysis of genes with a $p \leq 0.05$ (Tables S2 and S3). While some of the same programs are still enriched (cell adhesion, cell migration), we also identified new programs (Wnt signaling, splicing) that provide novel and relevant insights into the phenotypes (Fig. 6A).

As suggested, we have further validated up- and downregulated genes by qPCR, and the

© 2023. Published by The Company of Biologists under the terms of the Creative Commons Attribution License (https://creativecommons.org/licenses/by/4.0/).

results match our RNA-seq data (Fig. 6B). Due to the number of genes we wanted to assess, we chose qPCR over *in situ*. While qPCR does not give spatial information, we came up with a way to assess what cells of the developing kidney normally express our up- and downregulated genes of interest. We did this by assessing their enrichment in different cell types as generated from publicly available scRNA-seq data (Fig. 6C). We find that the up- and down regulated genes are enriched in cells of the stroma, smooth muscle, endothelium, and developing nephrons which aligns with our overall phenotypes and provides greater insights into what cell types are most affected by the deletion of *Ntn1* in the developing kidney. This data can be found in revised Fig. 6 and discussed in more detail in both the Results and Discussion section.

Minor criticisms:
1. Some of the figure numbers that appear throughout the manuscript do not match with the description, including "Fig 3c" on page 8 and "Fig 5d" on page 12.

Thank you, this has been amended in the revised manuscript.

Reviewer 2

An understanding of the molecular mechanisms controlling organ specific vascular patterning is essential for the generation of functional, stem cell derived tissues. However, this aspect of vascularization during embryogenesis lags far behind the mechanisms controlling vessel formation. Honneycutt et al. tackle this important, yet poorly understood process in the developing kidney.

The study is focused on the role of the guidance molecule,netrin-1, in murine kidney development. Netrin-1 is localized at both the mRNA and protein levels and ablated from the Foxd-1 stromal population by well-established genetic techniques. The resulting conditional netrin-1 null mice exhibit a complex renal phenotype but Honeycutt et. al do an excellent job in documenting it by quantitative and functional criteria. The study conclusively demonstrates that netrin-1 plays a direct role in controlling the temporal and spatial association of vascular mural cells to the renal vascular bed. Although the cause of the small kidney size cannot be attributed to a direct role for nephrin-1 in renal epithelial morphogenesis, the discussion of the manuscript well addresses different possibilities.

We are grateful for this reviewer's positive assessment of the manuscript and our findings. We have added additional data per the other reviewers' requests, in addition to expanded discussions of the phenotypes, and hope this reviewer agrees they have enhanced the manuscript.

Reviewer 3

Reviewer 3 Advance Summary and Potential Significance to Field

Honeycutt et al. investigate the role of the secreted guidance cue Netrin1 in the developing kidney. They used a stromal cell Cre line to generate mice harboring a stromal cell specific Ntn1 knockout, and showed that mutant mice develop hypoplastic kidneys at postnatal stages, but can live to adulthood and are described as healthy and devoid of renal defects. The developmental delay of the mutant kidneys is attributed to transient kidney nephrogenesis and arteriogenesis defects. The phenotypes presented overall match the ones shown in the accompanying manuscript by Luo et al, and support that stromal cell derived Netrin1 developmentally regulates morphogenesis of nephrons and renal arterial patterning and maturation. Mechanistically, the authors investigate one candidate Netrin1 receptor, Unc5c, which is expressed in nephron progenitors, but fail to observe defects in kidney development of these mutants. They also address potential redundancy of Netrin1 with Netrin3, but fail to observe any phenotypes in Ntn3 deficient mice. Finally, because they observe defective patterning of arteries and adjacent nerves, they address whether deleting TrkA dependent nerve growth affects vessel patterning in Ntn1 mutants, but find that ectopic arteries persist in Ntn1 mutants in the absence of axons, suggesting effects on the endothelium that remain however uninvestigated. Bulk RNA sequencing of wildtype and Ntn1 mutant kidneys is reported at E15, the genes regulated by Ntn1 deficiency are different from the ones reported in Luo et al, notably

© 2023. Published by The Company of Biologists under the terms of the Creative Commons Attribution License (https://creativecommons.org/licenses/by/4.0/).

KLF4 is not downregulated in this dataset. No validation of this dataset is provided. Overall, the data support important and complex developmental roles of Netrin1 in kidney morphogenesis, and provide a starting point for understanding the mechanism of action, which remains unclear.

We thank this reviewer for their critical assessment of our manuscript. We have addressed the concerns listed below and hope this has led to an improved manuscript with better understanding of the impacts of *Ntn1* deletion on kidney development.

Reviewer 3 Comments for the Author...
1- The delay in cessation of nephrogenesis seen in in Foxd1;Ntn1 kidneys is interesting but remains elusive. The authors claim that the delay in cessation of nephrogenesis seen in Foxd1;Ntn1 mice is independent of the Netrin1 receptor Unc5C. To make this claim, they should investigate Six2+ nephron progenitor cells in Unc5B KO kidneys at P4 and not at P0 where this population is still present in control kidneys. Also please show kidney size of Unc5c mutants at pre- and postnatal stages, and provide a table with the number of kidneys analyzed per genotype and per stage.

We appreciate the reviewer's suggestion and agree that a better assessment of the *Unc5c*$^{-/-}$ kidney size is warranted. We have included kidney length, body length, and body weight measurements at P0 and these are included in Fig. 3C. However, we do not find any significant differences in any of the measurements, which is in contrast to the significant difference in P0 kidney length observed in *Foxd1*$^{GC/+}$*; Ntn1*$^{fl/fl}$ animals. Since there are no significant size differences at P0, we did not believe this warranted investigation of *Unc5c*$^{-/-}$ kidney and body sizes at earlier stages.
The following has been added to the manuscript:

Pgs. 7-8: "We assessed the kidney phenotype of *Unc5c*$^{-/-}$ mice (Ackerman et al., 1997) and found no significant difference in kidney size compared to controls, in addition to no significant differences in animal body length and weight (Fig. 3B,C)."

In respect to the assessment of P4 kidneys, we agree this would be an excellent experiment. However, we are unfortunately unable to assess P4 kidneys. *Unc5c*$^{-/-}$ mice die between 24-48 hours on the C57Bl6/J background likely due to neurological problems (Susan Ackerman, personal communication-we have also never recovered homozygous adults from these crosses), and we have maintained all of our lines for this study on the C57 background. We did assess ~3 litters to determine if we could obtain any *Unc5c*$^{-/-}$ pups at P4 but unfortunately did not recover any. However, with the lack of any differences in kidney size, we predict there would be no significant delay in the cessation of nephrogenesis in these animals. The delay in cessation is a very rare kidney phenotype and is usually accompanied by hypoplasia/hyperplasia. If netrin-1 signals through Unc5c, there is likely some receptor redundancy in the nephron progenitors and would require the identification of these additional receptors and additional genetic crosses, which we felt was beyond the scope of this revision. The complexity of identifying all the putative receptors is noted in our Discussion. In reference to the inability to assess P4 animals, we have added the following to the manuscript:

Pg. 8: "The *Unc5c*$^{-/-}$ pups die within 24-48 hours of birth when maintained on the C57Bl6/J background, most likely due to neurological defects. Therefore, this precluded the analysis of P4 kidneys to determine whether nephrogenesis was extended. However, with the lack of any significant hypoplastic kidney phenotype in the *Unc5c*$^{-/-}$ mice, this suggests that netrin-1 signaling through Unc5c is not a major regulator of kidney development or that other receptors may functionally compensate."

2. Please explain rationale behind the TRKA deletion experiment in Fig.3. The text and its interpretation are hard to follow here.

We apologize that the rationale for this experiment was not clear. Netrin-1 acts as a guidance cue for both growing axons as they innervate tissue and blood vessels as they are forming in the tissue. The neurons/axons and endothelial cells can both express receptors for netrin-1 and respond accordingly (attraction or repulsion). Therefore, either axons or the vasculature (or both) could be responding to the netrin-1 guidance cues in the kidney. The axons tightly associate with the vasculature, specifically the smooth muscle coated endothelium, in the developing kidney. In the *Foxd1*$^{GC/+}$*; Ntn1*$^{fl/fl}$ kidneys, both axons and vessels appear to be mispatterned (Fig. 4C). Since the developing vasculature seems to 'lead' and the axons follow

© 2023. Published by The Company of Biologists under the terms of the Creative Commons Attribution License (https://creativecommons.org/licenses/by/4.0/).

the vessels, we wanted to confirm that the vasculature would still be mispatterned in the absence/reduction of nerves. Alternatively, if nerves would be the primary responder and 'leader', and vessels simply followed the axon pattern, then we would predict a restoration of normal vascular patterning in *Foxd1*$^{GC/+}$*; Ntn1*$^{fl/fl}$ kidneys (assuming the vessels are not dependent solely on netrin-1 for patterning). The deletion of *TrkA* reduces tissue innervation as this NGF receptor is required for survival of the neurons. In *TrkA* knockouts, the neurons die, and we see decreased innervation into the kidney. Despite the decrease in innervation, the blood vessels still form a normal pattern in *TrkA*$^{-/-}$ kidneys. However, when we delete *TrkA* and reduce innervation in *Foxd1*$^{GC/+}$*; Ntn1*$^{fl/fl}$ kidneys (*Foxd1*$^{GC/+}$*; Ntn1*$^{fl/fl}$*;TrkA*$^{-/-}$), the vascular patterning is still abnormal. This suggests that the vessels are not just simply following the axon pattern, but that the endothelium is missing the netrin-1 guidance cue and abnormally patterning even when innervation is diminished. We have modified the text as follows to hopefully clarify the reasoning behind this experiment:

Pg. 9: "As both axons and endothelium can respond to netrin-1 guidance cues, either may be mediating the neurovascular patterning. Therefore, we sought to differentiate the primary responder-axons or endothelium-to stromal-derived netrin-1 signals. Deletion of the *TrkA* receptor for Nerve Growth Factor (NGF) induces apoptosis of peripheral neurons such as those that innervate the kidney (Smeyne et al., 1994). We utilized the *TrkA* knockout to investigate whether kidney vascular patterning is disrupted in the setting of reduced kidney innervation (Liebl et al., 2000). In *TrkA* knockout kidneys, the endothelial (CD31) and arterial (□-smooth muscle actin, SMA) patterns were qualitatively normal at E16.5 (Fig. 4D). However, when combined with the *Foxd1*$^{GC/+}$*;Ntn1*$^{fl/fl}$ alleles to generate *Foxd1*$^{GC/+}$*;Ntn1*$^{fl/fl}$*;TrkA*$^{-/-}$ animals, the kidney endothelium still displayed abnormal patterning including looping and ectopic vessels (Fig. 4D). The ectopic and looping vessels were not associated with Tuj1+ axons (Fig. 4D arrowheads), suggesting the endothelium is the primary responder to netrin-1 guidance cues with nerves following the SMA+ vascular pattern as it is established."

3- While non-significant in their quantification, the 2month old Foxd1;Ntn1 KO kidney seems to have strongly decreased vascular density, and the image provided does not match their quantification.
Furthermore, Evans blue only highlights perfused vessel, can the authors co-stain with SMA and CD31 to analyze differences in vascular development.

We appreciate the reviewers' concern over the Evans blue images, and how well they represent the quantitative data. The control image in previous Fig. 4C (now 5C) appeared to have more density at the cortex on the right side of the kidney due to the arterial branches being less focused in this part of the image with increased background signal. While these Evans blue labeled adult kidney images may appear this way in the 3D reconstruction, Imaris software is able to distinguish the arterial branches and generate an accurate wireframe for analysis, thereby providing quantitative measurements. The complexity of labeling, clearing, and imaging whole adult organs means that not every image may look perfect, but through careful doublechecking of the Imaris analysis to ensure it is capturing the labeled vessels appropriately, we can be assured that the measured data is representative. Additionally, there is a range in variability in the 2 month samples, and the previous images may have represented the more extreme ends of variability. Nonetheless, we have replaced the control image with one that more clearly shows the cortical vascular labeling, now Fig. 5C.

We also appreciate the reviewers' concern of using Evans blue instead of SMA and CD31, as in the embryonic/postnatal studies. We utilized Evans Blue for the adult analysis due to the complexity and time-consuming nature of immunolabeling whole adult tissue. Additionally, it shows a preference for labeling arterial networks (Honeycutt and O'Brien, PMID: 33337254). The wholemount immunolabeling protocol can take up to a month to ensure full penetration and labeling with both primary and secondary antibodies (1-2 incubation weeks per antibody). We have also found the CD31 antibody becomes much less efficient at labeling in larger and adult tissue due to the significant density of endothelium in the tissue. We have tried (and tried again for this revision) to immunolabel whole adult tissues, but we find the variability in labeling efficiency does not present accurate representations of the vascular network. Therefore, we turned to a perfused dye to enable better labeling. While this method does come with its own limitations, we feel it is much better than what we can achieve by immunolabeling. We agree that it is only a proxy for the arterial network and not a direct comparison to the SMA+ CD31+

© 2023. Published by The Company of Biologists under the terms of the Creative Commons Attribution License (https://creativecommons.org/licenses/by/4.0/).

arterial vasculature. However, if vessels are perfused, they should be covered with mural cells such as a smooth muscle cell. If endothelium is not covered, it would generally keep proliferating as mural cells help stabilize the vessels. Also, if vessels are not actively perfused, they will regress. Therefore, altogether, the Evans blue perfused vessels should be a relatively accurate proxy for the SMA+ arterial network in adults, which is what we are comparing in Fig. 5C,D. While we were not able to efficiently label whole adult kidneys with SMA and CD31, we have added a supplemental figure, Fig. S5C, which shows sections of 3-month-old adult tissue (we were unable to obtain sufficient quality 2 month tissue during the revision time) labeled with SMA and CD146 (CD31 did not label the tissue well so we utilized CD146 which also marks endothelial cells). In the tissue sections provided, the SMA and CD146 labeling at the kidney cortex looks qualitatively similar between controls and *Foxd1*$^{GC/+}$;*Ntn1*$^{fl/fl}$ kidneys. However, it is incredibly difficult to assess vascular patterning and density in tissue sections lending support to the utility of our 3D analyses. The 3 month section data has been incorporated into the manuscript as follows:

Pg. 11: "At 2 months, quantification of *Foxd1*$^{GC/+}$; *Ntn1*$^{fl/fl}$ kidneys labeled with Evans blue revealed no significant differences in vascular metrics, although all three metrics trended towards a reduction (Fig. 5D). Tissue sections show the presence of qualitatively equivalent endothelial networks and SMA+ arteries between controls and *Foxd1*$^{GC/+}$; *Ntn1*$^{fl/fl}$ kidneys at 3 months (Fig. S5C)."

4- To validate bulk RNA sequencing data at least to some extent, please show if ectopic SMA positive cells are seen in the cortex of the mutant kidneys. It remains unclear if this phenotype is shared with Luo et al or not.

We appreciate the reviewer's suggestion of clarifying whether the phenotype is shared. Our bulk RNA-seq analysis was performed at E15.5 and at this stage we do not see any significant ectopic SMA+ cells on the surface of our kidneys (Fig. 5). This could be for several reasons. The first is that we are analyzing SMA at E15.5, a later stage than Luo, Gu et al. The ectopic SMA phenotype may have largely resolved in our animals by this time, if it was present. The second is how the labeling, clearing, and imaging is performed, and this is different between our studies and those of Luo, Gu et al. The staining protocols vary in the reagents used, we utilize different methods of clearing (iDISCO w/dibenzyl ether vs. 2:1 benzyl alcohol:benzyl benzoate), and we utilize a light- sheet microscope for imaging while Luo, Gu et al utilize a confocal microscope. The confocal microscope with higher resolution may be better at capturing details such as SMA on the surface of a kidney vs light-sheet which is lower resolution and better for capturing whole structures such as the vascular networks. Respectfully, we did not want to repeat what Luo, Gu et al have done at E13.5 to confirm if our phenotype matches. We feel this is a strength of our co-publications-that they complement each other with Luo, Gu et al focusing largely on earlier phenotypes and the initiation vs our studies which focus on later phenotypes and their persistence. Additionally, if we repeated our analyses at E13.5 using the methodology we have used throughout our paper, and did not see ectopic SMA, we wouldn't know if it was due to it being a real and significant difference between our studies or due to differences in labeling, clearing, and imaging as described above. We have further discussed this difference in the Discussion as presented here:

Pgs. 15-16: "Vascular patterning and maturity are both disrupted in the *Foxd1*$^{GC/+}$; *Ntn1*$^{fl/fl}$ kidneys in our study. **Luo, Gu et. al.** further define the maturity phenotype as being associated with a decrease in *Klf4* expression and accumulation of mural cells on the surface of the kidneys. We did not observe any obvious accumulation of mural cells at E15.5 or later stages (Fig. 5). This may be due to the ectopic accumulation resolving in our mice by E15.5, the earliest stage at which we assess SMA localization. Alternatively, our studies utilize different imaging modalities to assess the vascular networks. The confocal imaging utilized by Luo, Gu et al. provides higher resolution than our light-sheet, and may better capture the surface accumulation of SMA+ cells. Whether Klf4 is associated with the phenotype in our studies is unknown. We did not find a difference in *Klf4* expression in our E15.5 RNA-seq (Table S1), although this is 2 days later than the RNA-seq performed by **Luo, Gu et. al.** and *Klf4* levels may have normalized by this time or differences are masked by the normal increase of Klf4 in other kidney cell types. Therefore, the observed maturation phenotypes may initiate at the earliest stages of arterialization, around E13.5, and prior to our analysis at E15.5. Our RNA-seq analyses point to additional players that may be downstream of initiating events, including altered TGF-□1 signaling and collagen deposition (Fig. 6), which could further influence or support the vascular phenotypes. The timing of phenotype onset and how it is perpetuated could explain the

© 2023. Published by The Company of Biologists under the terms of the Creative Commons Attribution License (https://creativecommons.org/licenses/by/4.0/).

differences between our study and that of **Luo, Gu et. al.,** highlighting the complementary nature of our analyses."

Lastly, we have reanalyzed our bulk RNA-seq data with greater stringency and further validated up- and downregulated genes by qPCR. We have also assessed what cells of the developing kidney normally express these genes by assessing their enrichment in different cell types as generated from publicly available scRNA-seq data. Altogether, these data have narrowed down the programs and cell types most affected by the deletion of *Ntn1* in the developing kidney. This data can be found in revised Fig. 6 and discussed in more detail in both the Results and Discussion section.

Second decision letter

MS ID#: DEVELOP/2023/201886

MS TITLE: Netrin-1 directs vascular patterning and maturity in the developing kidney

AUTHORS: Samuel E Honeycutt, Pierre-Emmanuel Y N'Guetta, Deanna M Hardesty, Yubin Xiong, Shamus L Cooper, Matthew J Stevenson, and Lori L O'Brien
ARTICLE TYPE: Research Article

I am happy to tell you that your manuscript has been accepted for publication in Development, pending our standard ethics checks.

Reviewer 1

Advance summary and potential significance to field

The article by Honeycutt et al. details how Netrin-1 plays an important role in vascular patterning and maturation in the developing kidney. The authors use a combination of genetic deletion and advanced imaging couples with molecular analysis to determine an important developmental role of Netrin-1 in the renal stromal cells. The concept of the vessels and the nerves interacting and that in this context it is the vessels that are leading the nerves is novel and significant. The spatial imaging of the vessels and the nerves is also a strength and interesting to the field.

Comments for the author

The authors have addressed all my critiques.

Reviewer 2

Advance summary and potential significance to field

Comments for the author

The authors have addressed my comments, and I am pleased to recommend publication.

© 2023. Published by The Company of Biologists under the terms of the Creative Commons Attribution License (https://creativecommons.org/licenses/by/4.0/).
